# Temporal control of progenitor competence shapes maturation in GABAergic neuron development in mice

**DOI:** 10.1038/s41593-025-01999-y

**Published:** 2025-07-08

**Authors:** Ann Rose Bright, Yana Kotlyarenko, Florian Neuhaus, Diana Rodrigues, Chao Feng, Christian Peters, Ilaria Vitali, Elif Dönmez, Michael H. Myoga, Elena Dvoretskova, Christian Mayer

**Affiliations:** 1 Max Planck Institute for Biological Intelligence, Martinsried, Germany; 2 Max Planck Institute of Neurobiology, Martinsried, Germany; 3 Biomedical Center, Ludwig-Maximilians-Universität München, Martinsried, Germany

**Keywords:** Cell fate and cell lineage, Genetics of the nervous system

## Abstract

Diverse types of GABAergic projection neuron and interneurons of the telencephalon derive from progenitors in a ventral germinal zone called the ganglionic eminence. Using single-cell transcriptomics, chromatin accessibility profiling, lineage tracing, birthdating, transplantation across developmental stages and perturbation sequencing in mouse embryos, we investigated how progenitor competence influences the maturation and differentiation of these neurons. We found that the temporal progression of neurogenesis shapes maturation competence in ganglionic eminence progenitors, influencing how their progeny progress toward mature states. By contrast, differentiation competence—defined as the ability of progenitors to produce diverse transcriptomic identities—was maintained throughout neurogenesis. Chromatin remodeling, together with a regulatory module composed of the transcription factor NFIB and its target genes, influenced maturation competence in late-born neurons. These findings reveal how transcriptional programs and chromatin accessibility govern neuronal maturation and the diversification of GABAergic neuron subtypes during neurodevelopment.

## Abstract (web summary)

Unlike cortical progenitors, ventral telencephalic progenitors retain the ability to generate diverse neuron types during neurogenesis. Here, the authors show that ventral telencephalic progenitor maturation is gated by developmental timing, revealing a distinct regulatory logic underlying the development of inhibitory neurons.

## Main

**Fig. 1:**
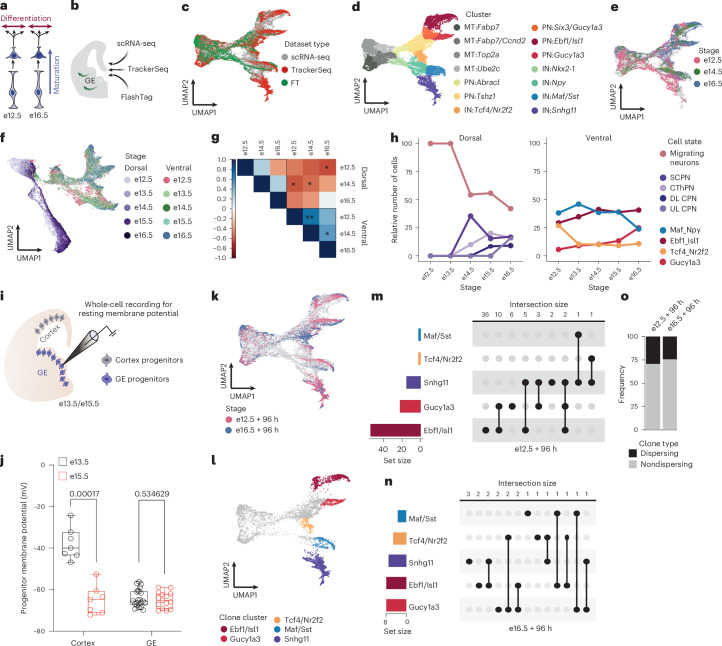
Stable differentiation competence in progenitors of GABAergic neurons. **a**, Schematic illustrating the difference between maturation and differentiation. **b**, Summary of methods used to investigate the competence of progenitors located in the GE. **c**, UMAP plot showing single cells derived from scRNA-seq, TrackerSeq and FT datasets aligned in Monocle3 (*n* = 41,460 cells; *n* = 40 embryos). Colors correspond to dataset type. **d**, UMAP plot showing single cells derived from scRNA-seq, TrackerSeq and FT datasets, with each color representing a different cluster. **e**, UMAP plot showing scRNA-seq datasets (*n* = 25,297 cells; *n* = 20 embryos), with colors indicating various collection stages. **f**, UMAP plot showing single cells from ventral (GABAergic lineage) and dorsal (glutamatergic lineage) telencephalon (*n* = 75,431 cells), with colors indicating various collection stages. **g**, Pearson’s correlation plot between dorsal and ventral progenitors at different developmental stages (*P < 0.05, **P < 0.01; two-sided *t*-test). **h**, Lineplot showing relative cell number of dorsal (left) and ventral (right) postmitotic neuronal states across stages. The annotation of dorsal cell states is derived from the original publication. **i**, Schematic illustrating whole-cell recording for resting membrane potential. **j**, Boxplots showing membrane potential in cortical and GE progenitors at e13.5 and e15.5; significance was tested using two-sided, unpaired *t*-tests (Cortex, *n* = 14; GE, *n* = 37) with false discovery rate correction. The median and 25th and 75th percentiles are represented as middle line and border lines of boxplots, with whiskers indicating the minimum and maximum value. **k**, UMAP plot showing TrackerSeq barcoded cells (*n* = 9,938), each color representing a stage of IUE; IUE at e12.5 and e16.5; scRNA-seq after 96 h. **l**, UMAP plot showing cell states at the branches used for clone grouping. **m**, Upset plot showing clonal intersections in TrackerSeq_e12.5+96h_. **n**, Upset plot showing clonal intersections in TrackerSeq_e16.5+96h_. **o**, Barplot showing the frequency of dispersing and nondispersing clones in TrackerSeq_e12.5+96h_ and TrackerSeq_e16.5+96h_. IN, interneuron; MT, mitotic; PN, projection neuron. Source data

The generation of diverse neuronal types is precisely coordinated in space and time^1^. This coordination relies on the competence of neuronal progenitors, shaped by gene regulatory programs that govern differentiation and maturation^2^. Here, we dissect two key aspects of progenitor competence: maturation competence and differentiation competence (Fig. 1a). Maturation competence, as defined here, refers to the potential of progenitors to generate postmitotic progeny with different maturation states, whereas differentiation competence refers to their potential to produce distinct neuronal subtypes. How these facets of progenitor competence are regulated and coordinated during neurogenesis is not fully understood.

Excitatory neurons of the mammalian cerebral cortex arise from proliferative zones in the dorsal telencephalon. The differentiation competence of their progenitors changes gradually during neurogenesis, influencing the temporal sequence of neuron subtype production^3–5^. Less is known about how progenitor competence shapes the development of inhibitory neurons originating from the ganglionic eminences (GEs) in the ventral telencephalon. During neurogenesis, mitotic progenitors in the ventricular zone of the GE divide to produce postmitotic precursors. These precursors initiate maturation and differentiation in the GE, and continue both processes as they migrate through the telencephalon and integrate into developing neural circuits^6^. Although there has been progress in correlating gene expression dynamics with chromatin accessibility^7^, the impact of mitotic progenitor competence on the differentiation and maturation of inhibitory neurons remains unclear.

Here, we explored the role of progenitor competence in forebrain inhibitory neuron development using a range of techniques, including FlashTag (FT) birth labeling, perturbation sequencing and clonal analysis. In contrast to progenitors of excitatory neurons in the cortex, progenitors in the GE maintained their differentiation competence to generate a consistent set of postmitotic cell states, as demonstrated by lineage tracing and the comparison of isochronic cohorts of early- and late-born inhibitory neurons. However, early- and late-born cohorts differed in the rate at which they progressed through maturation. This stage-specific difference in maturation competence originated from variations in chromatin accessibility profiles, as demonstrated by enhancer-driven gene regulatory networks (eGRNs). NFIB was identified as a key transcription factor (TF) in regulatory modules active in late-born progenitors, probably driving the observed changes in competence, as confirmed by perturbation sequencing and cleavage under target and release under nuclease (CUT&RUN) experiments. Finally, heterochronic transplantations revealed that maturation competence is influenced by the extrinsic environment. We provide an interactive web-based resource for exploring single-cell RNA sequencing (scRNA-seq), single-cell assay for transposase-accessible chromatin with high-throughput sequencing (scATAC-seq), CUT&RUN and eGRN datasets, which enables comparisons between GE- and cortex-derived neurogenesis (https://mayerlab.net/mouse-inhibitory-neuron-development/). Our findings demonstrate that both maturation and differentiation competence of progenitors are key determinants of neuronal development, playing distinct roles in shaping dorsal and ventral neuronal lineages.

## Results

**Extended Data Fig. 1:**
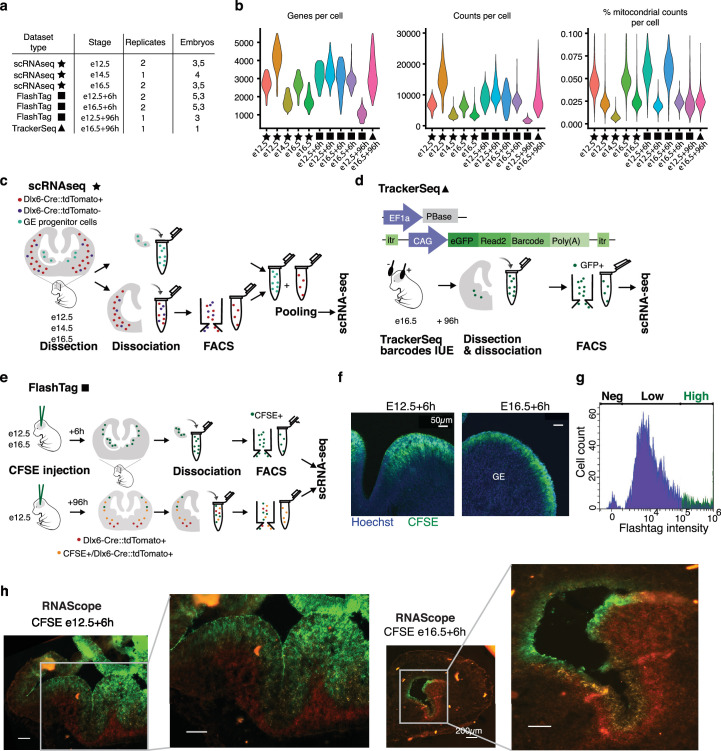
Experimental workflow and dataset characteristics. **a**, Table with dataset type, collection stage, number of replicates, and number of collected embryos per replicate. **b**, Violin plot illustrating the number of genes per cell, counts per cell and mitochondrial gene fraction per cell for each replicate. **c**, Schematic representation of the experimental procedure to generate the scRNAseq datasets. **d**, Schematic representation of the experimental procedure to generate the TrackerSeq datasets. **e**, Schematic representation of the experimental procedure to generate FlashTag datasets. **f**, FT^+^ cells, injected with CFSE. Injection at e12.5 and e16.5, collection after six hours; coronal sections of ganglionic eminences (GE) (n=3). **g**, FACS plot showing high-intensity FT^+^ cells. **h**, Coronal sections of the GE at e12.5 (left) and e16.5 (right). Cells are labelled with CFSE (in green), RNAscope hybridization probes for Ascl1 (in yellow), and for Gad2 (in red) (n=1). Source data

To investigate how progenitor competence influences neuronal maturation and differentiation in GABAergic lineages, we analyzed neuronal populations generated at different stages of neurogenesis (embryonic day (e), e12.5–e16.5) using distinct approaches, including scRNA-seq, barcode lineage tracing^8^ and fluorescent birthdating^9^ (Fig. 1a,b, Extended Data Fig. 1a,b and Supplementary Fig. 1a,b).

For scRNA-seq, we collected embryos from Dlx5/6-Cre::tdTomato mice at e12.5, e14.5 and e16.5, in which GABAergic neurons are labeled with a fluorescent reporter^10^. From the same brains, cortical and striatal regions were dissected manually and dissociated, and tdTomato-positive (tdTomato^+^) cells were enriched by fluorescence-activated cell sorting (FACS). Cells from the GE (without FACS enrichment) and tdTomato^+^ cells from the cortex and striatum (with FACS enrichment) were pooled to capture developmental states ranging from mitotic progenitors to postmitotic precursors and subjected to scRNA-seq (Extended Data Fig. 1c).

For barcode lineage tracing, we devised a published method called TrackerSeq, which uses heritable DNA barcodes to label individual progenitors and their progeny followed by multiplexed scRNA-seq^8,11^. We targeted progenitors in the GE at e16.5 with TrackerSeq plasmids via in utero electroporation (IUE), FACS-enriched electroporated cells 96 h later, and performed scRNA-seq (TrackerSeq_e16.5+96h_). In our analysis, we also included a published TrackerSeq dataset, in which TrackerSeq plasmids were electroporated at e12.5, and the targeted cells were collected 96 h later^8^ (TrackerSeq_e12.5+96h_; Extended Data Fig. 1d).

For birthdating, we used a technique called FlashTag (FT), which labels isochronic cohorts of cells with the fluorescent dye carboxyfluorescein succinimidyl ester (CFSE)^9^. In this method, mitotic cells layering the ventricle are labeled during the M phase of the cell cycle and maintain high fluorescence when leaving the cell cycle. We injected CFSE into the ventricles of e12.5 and e16.5 wild-type embryos. At 6 h later, we anatomically dissected the GE, FACS-enriched CFSE-labeled (FT^+^) cells and performed scRNA-seq (FT_e12.5+6h_ and FT_e16.5+6h_, respectively) (Extended Data Fig. 1e). At the time of cell collection for scRNA-seq (FT_e12.5+6h_ and FT_e16.5+6h_), coronal sections revealed FT^+^ cells in the ventricular zone and subventricular zone^9^, representing isochronic cohorts transitioning through mitotic progenitor, intermediate progenitor (*Ascl1*) and postmitotic precursor states (*Gad2*), as shown by RNAscope (Extended Data Fig. 1f–h). We also injected CFSE into the ventricles of e12.5 Dlx5/6-Cre::tdTomato mouse embryos, and collected FT^+^ cells 96 h later from anatomically dissected cortical and striatal tissues after their migration. TdTomato^+^ and FT^+^ cells were enriched by FACS and scRNA-seq was performed (FT_e12.5+96h_).

**Extended Data Fig. 2:**
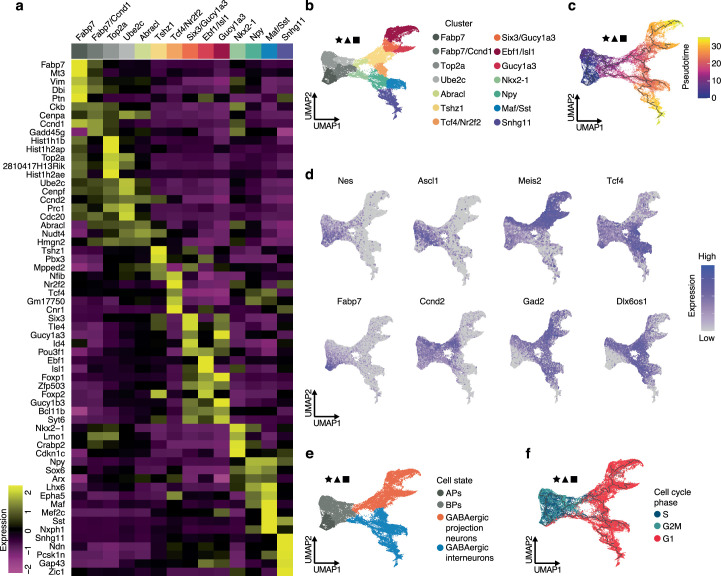
Cell state characterization in the GABAergic lineage. **a**, Heatmap of top five differentially expressed genes in GABAergic cell clusters. **b**, UMAP plot of combined datasets with cells colored by cluster identity. Clusters are annotated by one or two top marker genes. **c**, UMAP plot of combined datasets with inferred Monocle 3 trajectory. Cells are colored by pseudotime. **d**, Expression of marker genes in the combined dataset. *Nes* and *Fabp7* label APs, *Ascl1* and *Ccnd2* are markers for BPs. Post-mitotic inhibitory neurons express *Gad2* and *Dlx6os1*. *Meis2* labels PNs and *Tcf4* labels INs. **e**, UMAP plot of combined datasets, with cells colored by broad cell states. **f**, UMAP plot of combined datasets colored by cell cycle phases. Source data

We preprocessed and merged datasets from all three methods using Seurat^12^, aligned the batches using Monocle3 (ref. ^13^), and projected the data into a low-dimensional uniform manifold approximation and projection (UMAP) space (Fig. 1c). We then performed clustering (Fig. 1d and Extended Data Fig. 2a,b) and trajectory analyses using Monocle3 (Extended Data Fig. 2c), which learns the sequence of gene expression changes and uses a diffusion pseudotime algorithm to identify developmental trajectories. Consistent with previous work, clusters and trajectories represented a continuum of cell state transitions during cellular maturation and differentiation^6^^,8^ (Extended Data Fig. 2d). We manually annotated clusters based on marker gene expression, identifying them as mitotic apical progenitors (APs; *Fabp7*), mitotic basal progenitors (BPs; *Fabp7*, *Ccnd1*, *Top2a* and *Ube2c*), GABAergic projection neuron precursors (*Abracl*, *Tshz1*, *Six3*, *Gucy1a3*, *Ebf1* and *Isl1*) and GABAergic interneuron precursors (*Nkx2-1*, *Npy*, *Maf*, *Sst* and *Snhg11*; Fig. 1d and Extended Data Fig. 2e). After cell cycle exit, a common trajectory diverged, giving rise to distinct precursor states of projection neurons and interneurons. Each trajectory subsequently diverged into several postmitotic precursor states, which previous studies suggested are committed to broad inhibitory neuron types but have yet to fully mature and differentiate into specific subtypes^6,8^ (Extended Data Fig. 2e,f, Supplementary Fig. 2a,b and Methods).

To dissect neuronal maturation and differentiation during early stages of development, we first explored the scRNA-seq data in our combined single-cell trajectory (Fig. 1e) and calculated the sequential patterns of gene expression along the Monocle3 pseudotime trajectory. Surprisingly, the dynamic expression of TFs along the pseudotime trajectory was highly conserved across different stages of neurogenesis (e12.5, e14.5 and e16.5; Supplementary Fig. 2c).

### Dorsal–ventral differences in differentiation competence

The developmental progression described above differs from that observed in dorsal lineages^3,4^. To investigate this, we focused on differences in neurogenesis between dorsal and ventral lineages. We merged the scRNA-seq datasets generated in this study with published datasets of GABAergic neurons from e13.5 and e15.5 (ref. ^8^), as well as glutamatergic neurons from e12.5 to e16.5 (ref. ^3^) using Monocle3 (Fig. 1f and Supplementary Fig. 3a). In the UMAP representation, dorsal and ventral lineages overlapped at the level of APs but separated into distinct trajectories at the level of BPs and postmitotic precursors (Supplementary Fig. 3b). When comparing successive developmental stages, cells of the dorsal lineage showed a sequential shift in the UMAP positioning, consistent with findings from previous studies^3,14,15^. In contrast, cells of the ventral lineage overlapped across developmental stages (Fig. 1f). We identified genes associated with the emergence of inhibitory and excitatory neurons by selecting dynamic genes across pseudotime in the two lineages (Methods). Only few genes overlapped between inhibitory and excitatory lineages, primarily at the initial pseudotime scores (Supplementary Figs. 3c,d and 4a–d).

To quantify the temporal progression of dorsal and ventral progenitors, we calculated Pearson correlation coefficients between APs from each group, using highly variable genes (Methods). Ventral progenitors showed higher correlation coefficients between successive stages of neurogenesis than dorsal progenitors, indicating less change in their gene expression profiles (Fig. 1g and Supplementary Fig. 3e). Furthermore, differential gene expression analysis of ventral progenitors across stages revealed that only a few genes were upregulated at later stages of neurogenesis (Supplementary Fig. 5a). Genes that were downregulated at later stages were related primarily to self-renewal (Supplementary Fig. 5a,b), in line with a change in balance between cell proliferation and differentiation during neurogenesis^16^. Next, we annotated postmitotic cells based on marker gene expression (ventral lineage) or published data (dorsal lineage) and quantified the proportion of cells in postmitotic precursor states across different developmental stages (Supplementary Fig. 5c). Whereas the relative distribution of precursor states was similar across stages in ventral cells, it sequentially shifted in dorsal cells (subcerebral projection neuron (SCPN) → corticothalamic projection neuron (CThPN) → deep-layer callosal projection neuron (DL CPN) → upper-layer callosal projection neuron (UP CPN); Fig. 1h). Furthermore, we observed a similar trend when using fine-grained cluster annotation, as inferred from the integrated dataset (Supplementary Fig. 5d,e).

In progenitors of the cortical ventricular zone, bioelectrical processes have been shown to coordinate the temporal progression of progenitor competence, despite these cells being nonexcitable^5^. Specifically, progressive membrane hyperpolarization between e12.5 and e15.5 regulates the timing of progenitor competence and contributes to neuronal diversity. Given our observation that ventral GE progenitors maintain stable differentiation competence throughout neurogenesis, we asked whether this stability occurs despite similar bioelectrical changes or, alternatively, due to a lack of such changes. To distinguish these possibilities, we performed whole-cell patch-clamp recordings comparing membrane potentials in cortical and GE progenitors at e13.5 and e15.5 (Fig. 1i). Our recordings confirmed the progressive hyperpolarization in dorsal progenitors previously described in ref. ^5^, whereas membrane potentials in ventral progenitors remained stable over this developmental period (Fig. 1j and Supplementary Fig. 6a). This indicates fundamental differences in the mechanisms regulating progenitor competence between cortical and GE progenitors.

Together, our findings reveal several marked differences between dorsal and ventral progenitors. Whereas dorsal progenitors exhibit a temporal progression in differentiation competence and undergo hyperpolarization, ventral progenitors show more stable differentiation competence and unvaried membrane potential throughout neurogenesis, with GABAergic precursor states generated independently of developmental stages.

### Clonal divergence is maintained across neurogenesis

Our results so far suggest that, at the population level, progenitors in the GE can give rise to a similar set of precursor states throughout neurogenesis. To investigate whether the clonal progeny of individual progenitors can diverge into distinct precursor states, we next analyzed the barcode lineage tracing data in our combined dataset (Fig. 1k). We selected multicellular clones—clones containing several cells derived from a single progenitor—with cells located at the branch tips of the Monocle3 trajectory, where branch tips represent distinct developmental endpoints of the differentiation path (Fig. 1l–n and Supplementary Fig. 7a–d). We then grouped these clones based on whether their members were located within a single branch tip (nondispersing clones) or across several branch tips (dispersing clones). Consistent with previous studies, a subset of the TrackerSeq_e12.5+96h_ clones dispersed into several branch tips^8,11^. Notably, a similar proportion of dispersing clones was found in TrackerSeq_e16.5+96h_ (Fig. 1m–o). The true proportion of dispersing clones is probably higher than observed, as TrackerSeq recovers clones only partially due to cell loss during sample preparation^8^.

Clonal resolution enables linking individual mitotic progenitor cells to the fate of their postmitotic progeny. We tested whether the transcriptome of mitotic cells correlates with the transcriptome of their postmitotic daughter cells (Methods). Mitotic progenitor cells from nondispersing clones did not show a stronger correlation with the transcriptomic profiles of their clonal progeny compared to randomly selected progenitor cells (Supplementary Fig. 8a–d).

Overall, single-cell clonal analysis indicates that progenitor cells maintained stable differentiation competence throughout neurogenesis, consistent with the population-level findings.

### Maturation differs between early- and late-born neurons

**Fig. 2:**
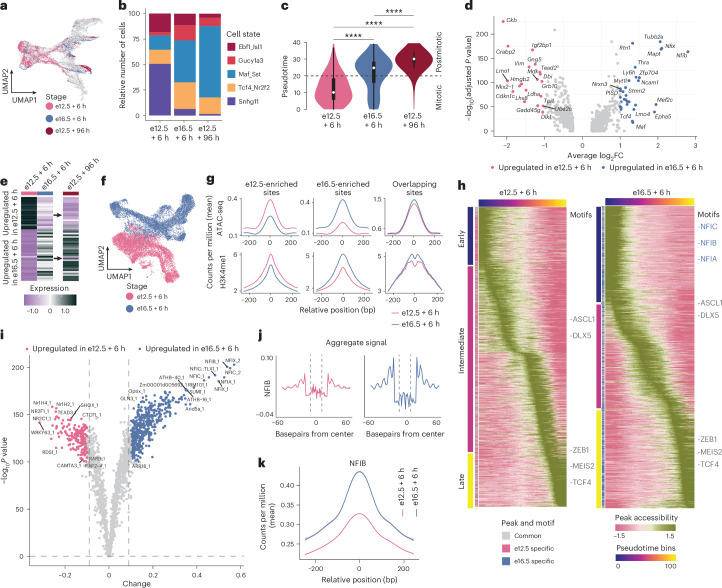
Timing of neurogenesis influences maturation competence. **a**, UMAP plot showing FT datasets (*n* = 6,225 cells; *n* = 19 embryos) colored by injection and collection stage; injection at e12.5 and e16.5, scRNA-seq after 6 h or 96 h. **b**, Barplot showing relative cell number of postmitotic neuronal states in FT_e12.5 + 6h_, FT_e16.5 + 6h_ and FT_e12.5 + 96h_. **c**, Violin plots showing the distribution of FT^+^ cells along the combined pseudotime trajectory, displayed for each condition; two-sided, unpaired Wilcoxon rank-sum test (****P < 0.0001, (*P* = 3.46 × 10^−82^, 2.87 × 10^−255^, 1.06 × 10^−107^), *n* = 2,000). The central point within the plot represents the median, hinges represent 25th and 75th percentile and whiskers show hinges ± 1.5 × interquartile range. **d**, Volcano plot displaying differential gene expression in postmitotic cells of FT_e12.5+6h_ and FT_e16.5+6h_; |log_2_FC| > 1, Bonferroni-adjusted *P* < 0.05 using two-sided Wilcoxon rank-sum test. **e**, Heatmap showing average scaled expression of differential genes in FT_e12.5+6h_ and FT_e16.5+6h_ postmitotic cells; visualized in all FT^+^ conditions. **f**, UMAP plot showing scATAC-seq datasets (*n* = 23,647 cells; *n* = 19 embryos); FT injection at e12.5 and e16.5, followed by scATAC-seq after 6 h. **g**, Coverage plot displaying scATAC-seq and H3K4me1 signal intensity for peak categories. The *x* axis is relative position (basepairs) and the *y* axis shows average counts per million. **h**, Heatmap displaying the accessibility of CREs across pseudotime for FT_e12.5 + 6h_ and FT_e16.5 + 6h_. Peaks are divided into ‘early’, ‘intermediate’ and ‘late’ based on accessibility profiles along pseudotime bins. Overlapping peaks are annotated in gray and unique peaks are annotated by stage-specific colors. Gray, overlapping motifs; blue, unique motifs. **i**, Volcano plot displaying −log_10_(*P* value) (*y* axis) and differential binding score (*x* axis) of TFs. *P* values were calculated using the subsampling procedure as proposed in ref. ^38^. Each dot represents a motif. **j**, Aggregate footprint profiles of NFIB in FT_e12.5+6h_ and FT_e16.5+6h_. **k**, Coverage plot showing chromatin accessibility dynamics at NFIB footprint sites for FT_e12.5+6h_ and FT_e16.5+6h_ datasets. Source data

**Extended Data Fig. 3:**
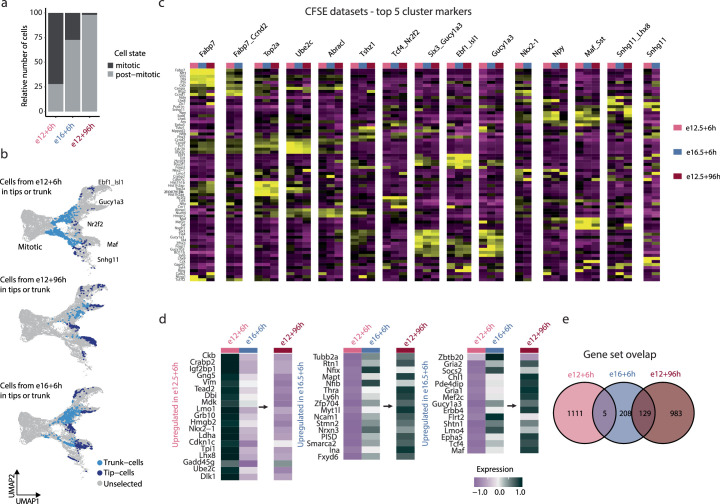
Comparison of early- and late-born cohorts. **a**, Barplot showing the cell ratio in different cell-cycle states for the FT_e12.5 + 6h_, FT_e16.5 + 6h_, and FT_e12.5 + 96h_ datasets. **b**, Post-mitotic cells of FT_e12.5 + 6h_, FT_e16.5 + 6h_, and FT_e12.5 + 96h_ highlighted in the UMAP-embedding of the merged dataset. Color indicates whether cells are part of branch-clusters or in post-mitotic trunk. Comparison of isochronic cohorts displayed side by side shows that cells from the FT_e12.5 + 6h_ cohort predominantly occupy intermediate positions, indicating progression toward the branch tip (Snhg11), whereas cells from FT_e16.5 + 6h_ and FT_e12.5 + 96h_ cohorts have reached the branch tips. **c**, Heatmap of marker gene expression for branch tips split by isochronic cohorts demonstrates that low-abundance states exhibit gene-expression profiles consistent with other cells at the branch tips, supporting their correct classification. **d**, Detailed heatmap of differentially expressed genes between FT_e12.5 + 6h_ and FT_e16.5 + 6h_; 1<log2FC<–1, Bonferroni-adjusted P<0.05 (two-sided Wilcoxon rank-sum test). Expression is visualized in FT_e12.5 + 6h_, FT_e16.5 + 6h_ and FT_e12.5 + 96h_ datasets. **e**, Venn diagram showing the intersection of FT_e12.5 + 6h_, FT_e16.5 + 6h_, and FT_e12.5 + 96h_ marker genes; FC > 0.25, Bonferroni-adjusted P < 0.05 (two-sided Wilcoxon rank-sum test). Source data

Next, we examined the maturation and differentiation of postmitotic cells at different stages of neurogenesis, using the fluorescent birthdating data in our combined single-cell trajectory (Fig. 2a). FT^+^ cohorts collected 6 h after CFSE application (FT_e12.5+6h_ and FT_e16.5+6h_) contained mitotic progenitors as well as early postmitotic neuronal precursors. At 96 h after CFSE application (FT_e12.5+96h_), FT^+^ cohorts contained exclusively postmitotic cells (Extended Data Fig. 3a), consistent with the notion that FT marks isochronic cohorts of cells that exit the cell cycle shortly after CFSE application^6,14^. The postmitotic fractions across all three conditions (FT_e12.5+6h_, FT_e12.5+96h_, FT_e16.5+6h_) included cells from the same precursor states, but with differences in their relative population sizes (Fig. 2b). The rarity of some states in our analysis probably reflects the varying maturation stages of isochronic cohorts at the time of capture. For example, cells in the FT_e12.5+6h_ cohort appear to be transitioning towards branch tips, as indicated by their intermediate positions on the UMAP embedding (Extended Data Fig. 3b). States with a low abundance of cells in a particular cohort shared consistent gene expression profiles with corresponding states in other cohorts (Extended Data Fig. 3c). Next, we quantified the Monocle3 pseudotime scores as a proxy for the degree of maturation acquired by the different FT^+^ cohorts. As expected, given its later collection, FT_e12.5+96h_ showed higher pseudotime scores than FT_e12.5+6h_. The pseudotime score of FT_e16.5+6h_ was markedly higher than that of FT_e12.5+6h_, even though both were collected after 6 h (Fig. 2c). Next, we performed a differential gene expression analysis between postmitotic cells of the 6-h cohorts (FT_e12.5+6h_ versus FT_e16.5+6h_; Fig. 2d). Genes upregulated in FT_e16.5+6h_ overlapped with those upregulated in FT_e12.5+96h_ (Fig. 2e and Extended Data Fig. 3d,e). The intersection analysis of cohort marker genes (Methods) further supported this result, revealing a higher overlap between FT_e16.5+6h_ and FT_e12.5+96h_ marker genes (Extended Data Fig. 3e). These findings suggest that late-born neurons reach a similar gene expression profile within 6 h as early-born neurons within 96 h. Many of the genes upregulated in FT_e16.5+6h_ were associated with the promotion of neuronal proliferation and migration (Supplementary Table 1). Some of these genes were linked specifically to neuronal signaling pathways. Overall, our results using FT birthdating suggest that, although newborn neurons at different stages transition into similar precursor states, the rate and extent of their maturation differ, with late-born neurons maturing more rapidly compared to early-born neurons.

The observed maturation shift in the production of GABAergic neurons during neurogenesis may help adapt newly born neurons to the varying time available for network integration between early- and late-born neurons. We tested this hypothesis using electrophysiological recordings at postnatal day 8, but were unable to definitively confirm or disprove it (Supplementary Results and Supplementary Figs. 21 and 22).

### Maturation shift correlates with chromatin accessibility

**Extended Data Fig. 4:**
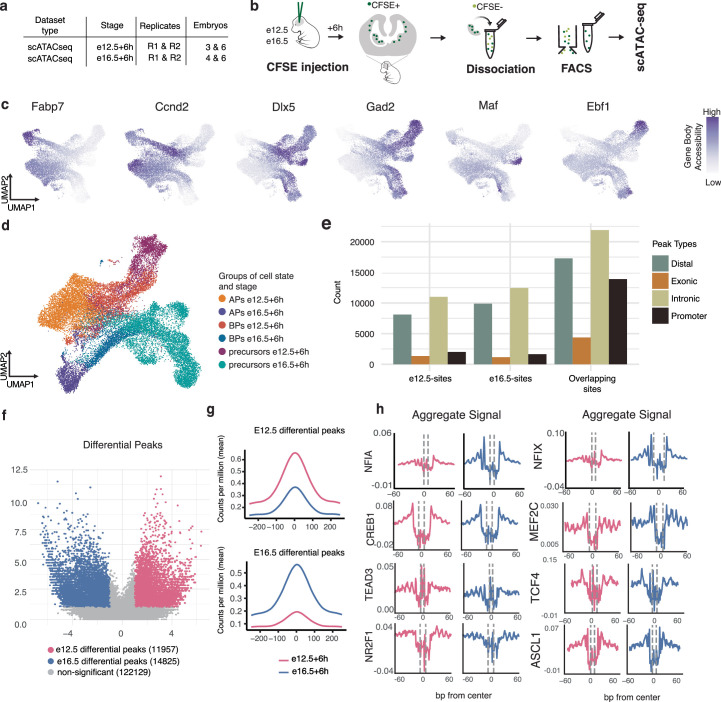
Transcription factor activity and chromatin remodeling in early and late cohorts. **a**, Overview of FT^+^ scATAC-seq datasets: collection stage, number of replicates, and number of embryos per replicate. **b**, Schematic representation of the experimental procedure to generate FT^+^ scATAC-seq datasets. **c**, UMAP depiction of gene body accessibility for marker genes. *Fabp7* for APs, *Ccnd2* for BPs, *Dlx5* and *Gad2* for postmitotic inhibitory neurons, *Maf* for INs and *Ebf1* for PNs. **d**, UMAP-embedding of cells in FT^+^ scATAC-seq datasets. Cells are grouped and colored by broad cell state and stage. **e**, Barplot quantifying peak types (distal, exonic, intronic, and promoter) at e12.5, e16.5, and overlapping sites. **f**, Volcano plot displaying differentially accessible peaks between stages, with x-axis showing fold-change and y-axis showing –log10(p-value). Two-sided Wilcoxon rank-sum test using FDR-correction. Significant TFs are highlighted (P <= 0.1 and 1<log2FC<–1. **g**, Coverage plots of e12.5-enriched sites (top) and e16.5-enriched sites (bottom). Aggregated coverage was calculated for each stage separately. **h**, Aggregate footprint profiles of select transcription factors in FT_e12.5 + 6h_ and FT_e16.5 + 6h_. Source data

To explore whether the different maturation dynamics we observed at embryonic stages are associated with changes at the chromatin level, we profiled chromatin accessibility using scATAC-seq^17^ on samples derived from FT^+^ cohorts in the GE (Extended Data Fig. 4a). We injected CFSE into the ventricles of e12.5 and e16.5 wild-type embryos, anatomically dissected the GE 6 h later (FT_e12.5+6h_, FT_e16.5+6h_, respectively), enriched FT^+^ cells via FACS and performed scATAC-seq (Extended Data Fig. 4b). Following sequencing, we mapped the paired-end reads to a reference genome and employed the ArchR framework^18^ for quality control, as well as data processing steps such as dimensionality reduction, clustering and peak calling. Cell annotations were determined based on gene body accessibility patterns of cell state marker genes (Fig. 2f and Extended Data Fig. 4c,d).

In contrast to the scRNA-seq experiments (Fig. 1e), the isochronic cohorts FT_e12.5+6h_ and FT_e16.5+6h_ in the scATAC-seq experiment occupied distinct regions on the UMAP plot, both in mitotic and postmitotic cell states (Fig. 2f and Extended Data Fig. 4d). To identify and quantify the *cis*-regulatory elements (CREs) responsible for this separation, we independently conducted peak calling on FT_e12.5+6h_ and FT_e16.5+6h_. We then categorized the resulting peaks to identify genomic sites with e12.5-enriched peaks, e16.5-enriched peaks and shared sites that were not stage-specific (‘e12.5 sites’, ‘e16.5 sites’ and ‘overlapping sites’, respectively; Supplementary Fig. 9a and Methods). Subsequently, we computed the scATAC-seq fragment distribution and displayed the results in coverage plots. At e12.5 sites, we observed higher accessibility in FT_e12.5+6h_ than in FT_e16.5+6h_. Conversely, e16.5 sites had higher accessibility in FT_e16.5+6h_ than FT_e12.5+6h_ (Fig. 2g). The peak sets were divided by genomic region into promoters, distal, exonic and intergenic regions (Extended Data Fig. 4e). At e12.5 sites and e16.5 sites, distal and intergenic regions represented the largest proportion of peaks. Together, this indicates that the chromatin accessibility undergoes marked changes between different stages of development, implying a dynamic process of chromatin remodeling that occurs predominantly at distal and intronic regions. To further categorize the identified sites as poised-active distal regulatory elements, we analyzed the distribution of H3K4me1 fragments, a well-established enhancer mark^19^, using forebrain chromatin immunoprecipitation followed by sequencing (ChIP–seq) data from ENCODE^20^. H3K4me1 profiles aligned closely with chromatin accessibility profiles (Fig. 2g). Specifically, e12.5 sites exhibited a stronger H3K4me1 signal at e12.5 compared to e16.5, and the contrary was observed for e16.5 sites. These observations suggest that distal regulatory elements are potentially maintained in a poised-active state and probably drive the stage-specific dynamics in chromatin accessibility.

To complement our earlier analysis (Fig. 2g), which identified e12.5- or e16.5-enriched sites, we performed a differential peak analysis (Methods). This analysis resulted in 11,957 peaks differentially accessible at e12.5, 14,825 peaks differentially accessible at e16.5 and 122,129 nonsignificant peaks (Extended Data Fig. 4f). To visualize changes in chromatin accessibility, coverage plots were generated, revealing trends consistent with those observed using the previous peak sets (Extended Data Fig. 4g), further validating the stage-specific changes in chromatin accessibility between e12.5 and e16.5.

To explore how the stage-specific accessibility of CREs relates to the maturation process, we used ArchR to assign a pseudotime score to cells, capturing their position along the maturation trajectory (from APs to BPs to precursor cells; Fig. 2h). We used ArchR to perform peak calling along the inferred trajectory and grouped the identified CREs into three main phases based on their accessibility profiles along pseudotime: ‘early’, ‘intermediate’, and ‘late’ CREs, corresponding broadly to APs, BPs and precursor cells. We found more peaks in early CREs at FT_e16.5+6h_ in respect to FT_e12.5+6h_, suggesting an early opening of additional regulatory elements in e16.5 progenitors (Supplementary Fig. 9b). To identify associated TFs, we subsequently conducted motif scanning on the peaks that were specific to the early, intermediate and late CREs at both stages. From this analysis, we identified both common and stage-specific motifs (Fig. 2h). Motifs of TFs associated with inhibitory neuron development, such as TCF4, MEIS2, EBF1 and ISL1 (Supplementary Fig. 2b), were detected at both stages. Conversely, several motifs from the nuclear factor I (NFI) family (NFIA, NFIB and NFIC) were linked exclusively to early CREs in FT_e16.5+6h_. The NFI TFs are known for regulating key steps during brain development^21^, such as neural and glial cell differentiation^22^, neuronal migration^23^ and maturation^24^.

DNA-binding proteins, like TFs, protect genomic regions from Tn5 integration during scATAC-seq sample preparation, creating a measurable ‘footprint’ that indicates the binding patterns of TFs on chromatin. These footprints thus predict the strength of TF binding (that is, TF activity) and binding locations. We conducted a footprint analysis on the FT^+^ cohorts, using TOBIAS^25^, and performed a differential binding analysis. Among the differential TFs, the NFI family showed a statistically significant increase of TF binding activity in FT_e16.5+6h_ (Fig. 2i). To visualize and evaluate this finding, we generated stage-specific aggregate footprint profiles for select TFs (Fig. 2j and Extended Data Fig. 4h). NFIX, NFIC and NFIA displayed TF activity only in FT_e16.5+6h_, whereas NFIB displayed TF activity already in FT_e12.5+6h_, which significantly increased in FT_e16.5+6h_ (Fig. 2j and Extended Data Fig. 4h). This aligns with the gradual increase in gene expression patterns of NFI family TFs observed in the transcriptomic data (Supplementary Fig. 9c). Next, to assess whether sites where NFI family TFs bind (footprint sites) exhibit dynamic changes in accessibility, we calculated the fragment distribution in these regions. Coverage plots displayed a temporal increase in accessibility from FT_e12.5+6h_ to FT_e16.5+6h_ at NFIB, NFIA, NFIC and NFIX footprint sites (Fig. 2k and Supplementary Fig. 9d). Our findings suggest a link between specific TFs and the observed chromatin dynamics, underscoring their potential role in chromatin remodeling.

Together, these findings demonstrate that isochronic FT^+^ cohorts exhibit stage-specific chromatin accessibility, driven mainly at CREs. Furthermore, the NFI family of TFs has a crucial role in characterizing FT_e16.5+6h_ cells based on their expression and early activation of regulatory elements.

### NFIB modulates the network underlying maturation competence

Our analysis of scATAC-seq profiles between FT_e12.5+6h_ and FT_e16.5+6h_ revealed that CREs, such as enhancers, are the primary source of heterogeneity. To infer enhancer-driven regulatory interactions, we applied SCENIC+ (ref. ^26^) to integrate scRNA-seq and scATAC-seq data from FT_e12.5+6h_ and FT_e16.5+6h_. This approach enables the identification of genomic binding events (that is, TFs binding to regulatory sites) and their links to downstream target genes. We grouped cells by collection stage (e12.5 and e16.5) and broad states (APs, BPs and precursors), obtaining six groups in total (Supplementary Fig. 10a). After running the SCENIC+ pipeline with standard filtering, the resulting eGRN contained 147 TFs that bound on average 168 sites, with each site regulating one to three target genes (mean, 1.1; Supplementary Fig. 10b–d). The activity of regulatory modules (that is, expression of TF and associated target genes) was scored in each cell using a previously established method^27^, and enriched modules for each group were identified (Supplementary Fig. 10e). Modules of canonical cell-state markers were enriched in their respective groups: Hes5, Hes1 and Pax6 modules in APs^28,29^; Ascl1 and Dlx2 modules in BPs^30,31^; and Dlx5 or Lhx6 modules in neuronal precursors^31,32^. We also found modules exhibiting patterns that were specific to certain cell states or developmental stages. For example, Nkx2-1 was active in BPs and precursor states, yet remained restricted to FT_e12.5+6h_. By contrast, modules of NFI family TFs were active across all cell states in FT_e16.5+6h_, with the highest activity in APs compared to BPs and precursor cells (Supplementary Fig. 10e).

**Fig. 3:**
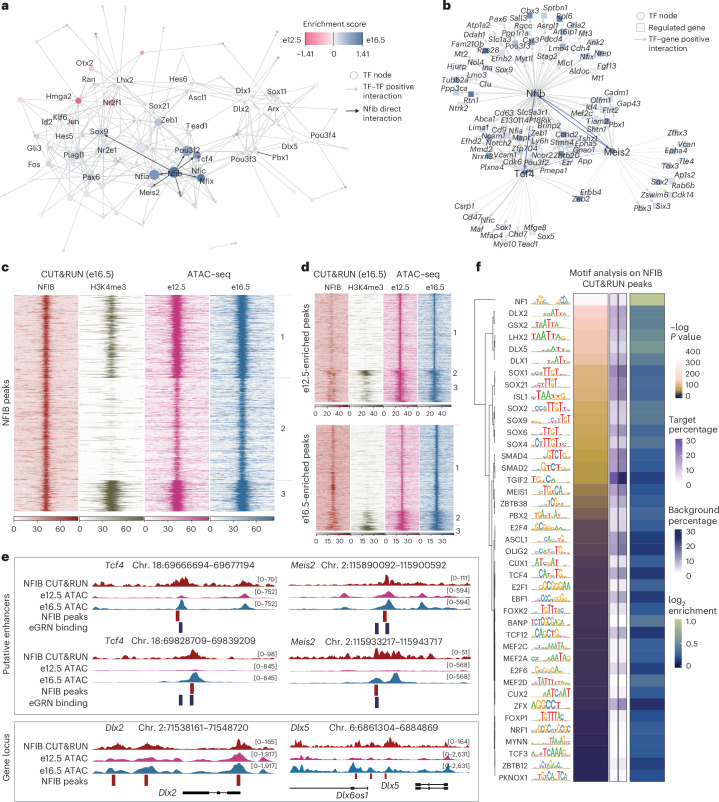
Nfib regulates a shift in gene regulatory programs. **a**, An eGRN graph displaying positive interactions between TFs active in APs. Node color indicates enrichment score by stage and node size indicates the number of direct targets per TF. Select TFs are annotated. Direct interactions originating from NFIB are highlighted. **b**, An eGRN subgraph highlighting downstream targets of Nfib, Tcf4 and Meis2 at e16.5. Nfib, Tcf4 and Meis2 nodes are indicated by node shape. Interactions between Nfib, Tcf4 and Meis2 are highlighted. Node color reflects the enrichment score by stage. **c**, Heatmap displaying signal enrichment of NFIB peaks across datasets: NFIB and H3K4me3 CUT&RUN at e16.5 GE, and scATAC-seq at e12.5 and e16.5. **d**, Heatmap displaying signal enrichment of e12.5- and e16.5-enriched peaks across datasets: NFIB and H3K4me3 CUT&RUN at e16.5 GE, and scATAC-seq at e12.5 and e16.5. **e**, Genome browser tracks of putative enhancer regions for *Tcf4* and *Meis2* and gene loci for *Dlx2* and *Dlx5*, featuring NFIB CUT&RUN and scATAC-seq at e12.5 and e16.5. **f**, Enriched TF motifs in NFIB CUT&RUN peaks. TFs are ordered by their *P* value (binomial test). For each TF, the motif logo, target- and background percentage and the resulting enrichment are shown. The dendrogram on the left shows the sequence similarity of motif logos. Source data

**Extended Data Fig. 5:**
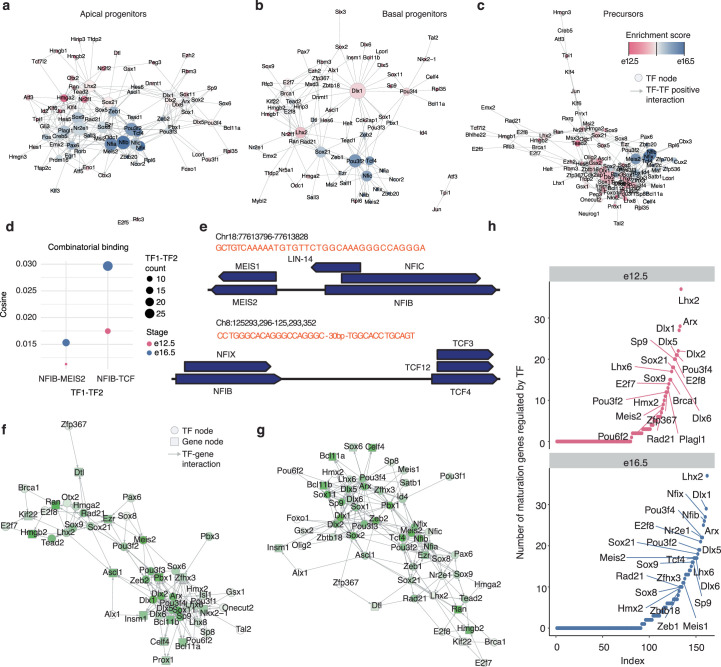
Transcription factor interaction analysis in subnetworks. Subnetwork for APs (**a**), BPs (**b**) and precursors (**c**). Each subnetwork is merged across e12.5 and e16.5, with node color indicating the difference in expression between stages. The size of nodes reflects the number of downstream targets per TF. Subnetworks show only interaction between TFs. **d**, Dot plot showing cosine score on the y-axis and TF pairs on the x-axis. Color and size indicate the stage and the number of occurrences of TF1-TF2, respectively. **e**, Genomic view of transcription factor binding sites for NFIB-MEIS2 and NFIB-TCF4. **f**, TF interaction network of TFs that regulate genes dynamic along the maturation trajectory in e12.5. Nodes are colored by their average expression at e12.5. **g**, TF interaction network showing transcription factors (TFs) regulating genes with dynamic expression along the maturation trajectory at e16.5. Nodes are colored based on their average expression levels at e16.5. **h**, Number of bound genes per TF (out-degree) at e12.5 and e16.5; subsetted for genes dynamic along the maturation trajectory and their upstream TFs. TFs with an out-degree higher or equal than eight are labelled. Source data

Next, we inferred active gene regulatory interactions specific to the six groups by filtering the eGRNs for modules active in over 50% of cells within each group and applying an additional filter on the target genes based on expression level (Methods). We obtained six subnetworks, each containing state and stage-specific modules of active TFs and target genes. We compared subnetworks of APs, BPs and precursors across stages to infer dynamic modules and the regulatory interactions between them (Methods). Specifically, we focused on subnetworks of APs to identify modules that maintain or modulate progenitor competence. Modules of canonical inhibitory neuron markers, such as Dlx1, Dlx2 and Arx^31,33^, were maintained throughout both stages, whereas modules linked to progenitor self-renewal, such as Hmga2, Nr2f1 and Nr2f2 (refs. ^34,35^), were enriched in e12.5 APs (Fig. 3a). APs at e16.5 were characterized by enriched activity of Nfib, together with Nfia, Nfix, Pou3f2, Meis2 and Tcf4. In line with previous studies, NFIB acts as an upstream regulator of NFIX^36^, but also as an upstream regulator of NFIA, POU3F2, MEIS2 and TCF4 (Fig. 3a). The NFIB-led regulatory module was consistently enriched in BPs and precursors at e16.5 (Extended Data Fig. 5a–c), suggesting a role of NFIB as a central regulator.

Of particular interest to us were the interactions between NFIB with MEIS2 and TCF4, which are TFs specific to the development of inhibitory projection neurons and interneurons, respectively^11,37^. Moreover, these TFs share common direct target genes in different cell states of FT_e16.5+6h_ (Supplementary Fig. 11a), suggesting combinatorial binding of NFIB with TCF4 or MEIS2. To test this hypothesis, we used TF-COMB—a tool for identifying enriched TF binding motifs in chromatin accessibility data^38^—to analyze peaks from FT_e12.5+6h_ and FT_e16.5+6h_ scATAC-seq datasets. Interestingly, NFIB was found to collaborate with these TFs at both stages, with higher cosine scores and increased binding events for NFIB–TCF4 and NFIB–MEIS2 in e16.5 peaks, suggesting a stage-specific enhancement of regulatory interactions that may drive late-stage maturation processes (Extended Data Fig. 5d,e). Next, using SCENIC+, we identified direct downstream target genes shared between NFIB, MEIS2 and TCF4 (Fig. 3b). Gene ontology enrichment analysis of these downstream genes revealed roles in brain development, neuron fate specification and the positive regulation of cell proliferation (Supplementary Fig. 11b). We then identified a group of genes exhibiting dynamic expression across the maturation trajectory and inferred their upstream TFs in FT^+^ cohorts, sorting TFs by the number of regulated maturation genes. Temporally conserved TFs such as DLX1 and LHX2, along with e16.5-specific TFs like NFIB and NFIX, regulated the largest number of genes, further supporting our previous observations (Extended Data Fig. 5g–h). To assess the functional relevance of the e12.5- and e16.5-enriched peaks, we quantified the proportion of these peaks that are contained in the eGRN. This analysis revealed substantial overlap: 71.43% of e12.5-enriched peaks and 70.93% of e16.5-enriched peaks were predicted to be part of TF–enhancer–target gene interactions (Supplementary Fig. 11c), suggesting that most peaks are likely to have functional relevance.

Next, we performed CUT&RUN on unfixed, dissociated cells from the GE of e16.5 embryos using an NFIB antibody, with IgG and H3K4me3 as controls, to identify and validate genomic targets of NFIB in vivo. Mapping and sample processing were carried out using established tools and pipelines (Methods). MACS2 peak calling identified approximately 21,000 narrow peaks (*P* value cutoff of 1 × 10^−4^) corresponding to NFIB binding relative to the IgG control. To investigate the relationship between NFIB binding and chromatin accessibility during development, we plotted signal intensities at NFIB-binding sites for NFIB, H3K4me3 and FT_e12.5+6h_ and FT_e16.5+6h_ scATAC-seq datasets (Fig. 3c). *K*-means clustering of these binding sites revealed three distinct clusters, all characterized by strong NFIB binding. Cluster 2 lacked H3K4me3 enrichment, suggesting these regions may represent nonpromoter elements with increased chromatin accessibility at e16.5 relative to e12.5 (Supplementary Fig. 12b). By contrast, clusters 1 and 3 showed intermediate to high levels of H3K4me3, indicating that many of these regions are promoters. To further examine NFIB binding at temporally dynamic peaks, we compared NFIB and H3K4me3 signal intensities at e12.5- and e16.5-enriched sites identified in our scATAC-seq data. NFIB binding was significantly higher at e16.5-enriched sites, whereas e12.5 sites showed markedly lower or no signal (Fig. 3d and Supplementary Fig. 12c,d). These findings support our hypothesis that NFIB is associated with chromatin remodeling at e16.5.

We validated predicted eGRN interactions of NFIB (for example, NFIB–*Tcf4*, NFIB–*Meis2*) by confirming NFIB binding at predicted enhancers (Fig. 3e). Additionally, we observed NFIB binding at promoters of TFs involved in inhibitory neuron development, such as *Dlx2* and *Dlx5* (Fig. 3e). Furthermore, we quantified the fraction of eGRN predicted target regions of NFIB that was validated by NFIB CUT&RUN, by calculating the fraction of target regions with a binding event (43.7%). Motif analysis of NFIB peaks using HOMER (ref. ^39^) displayed significant enrichment of additional TF motifs associated with inhibitory neuron development including DLX1/2/5, ISL1, SOX2, ASCL1, MEIS1/2 and TCF4 (Fig. 3f).

In summary, we observed gene regulatory interactions that drive cell state- and stage-specific dynamics, with NFIB playing a leading role in late-born progenitors through direct and combinatorial binding at genes involved in maturation and differentiation.

### Influence of extrinsic environment on maturation competence

**Fig. 4:**
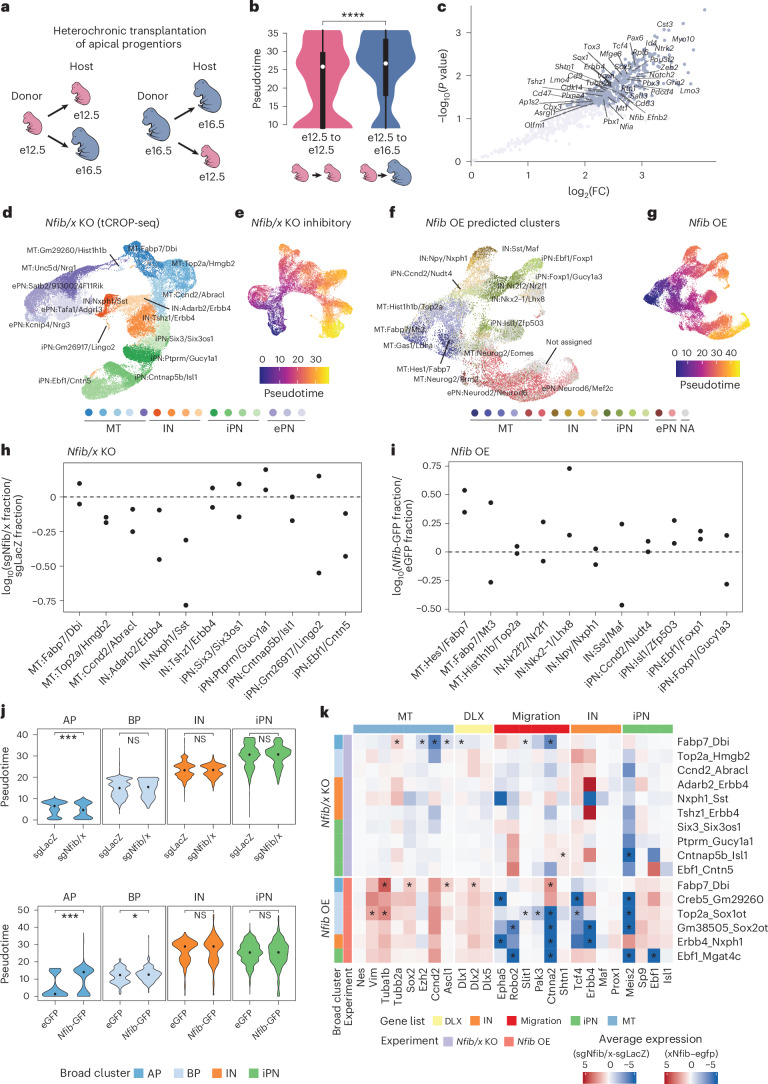
Intrinsic and extrinsic factors regulating progenitor competence. **a**, Schematic overview of donor and host stages for homo- and heterochronic transplantation experiments. **b**, Distribution of transplanted cells along pseudotime in AP_e12.5 → e12.5_ and AP_e12.5 → e16.5_; two-sided Wilcoxon rank-sum test (****P < 0.0001, *P* = 6.02 × 10^−14^; *n* = 1,000). Boxplots indicate median as point, 25th and 75th percentiles as hinges and hinges ± 1.5 × interquartile range as whiskers. **c**, Differentially expressed genes between AP_e12.5 → e12.5_ and AP_e12.5 → e16.5_; 1 < log_2_FC < −1; Benjamini–Hochberg corrected *P* < 0.05 (two-sided Wald test). Only genes downstream of NFIB, MEIS2 and TCF4 are labeled. **d**, UMAP embedding of cells collected in *Nfib/x* knockout (KO) (*n* = 47,079 cells; *n* = 10 embryos). Cells are annotated by broad cell state and the cluster’s top two marker genes. **e**, UMAP embedding of subsetted inhibitory neuron precursors and their progenitors in *Nfib/x* KO (n = 32,783). Cells are colored by inferred pseudotime scores. **f**, Cells from *Nfib* overexpression (OE) shown in UMAP embedding (*n* = 30,019 cells; *n* = 7 embryos). Cell labels were predicted using label transfer. Cells with low prediction score are labeled as not assigned. **g**, UMAP embedding of cells in *Nfib* OE. Cells are colored by inferred pseudotime scores. **h**, Proportion change per cluster in *Nfib/x* KO. For each biological replicate, the fraction of cells containing sgNfib/x was compared to the fraction of cells containing sgLacZ. **i**, Proportion change per predicted label in *Nfib* OE. For each biological replicate, the fraction of cells containing *Nfib*-GFP plasmid was compared to the fraction of cells containing eGFP control plasmid. **j**, Distribution of pseudotime scores between conditions across broad cell states in *Nfib/x* KO (top) and *Nfib* OE (bottom). Dot shows median of corresponding distribution. Two-sided Wilcoxon rank-sum test, ***P < 0.001, **P < 0.01, *P < 0.05, (*P* = 1.64 × 10^−08^, 2.75 × 10^−11^, 0.02) for *Nfib/x* KO AP, *Nfib* OE AP and BP, *Nfib/x* KO, *n* = 2,205/5,592/5,350/12,768 for AP/BP/IN/iPN; *Nfib* OE, *n* = 2,025/1,452/545/2,354 for AP/BP/IN/iPN. **k**, Change in gene expression upon perturbation for selected genes. Average gene expression was calculated per cluster and condition. Expression change was calculated by dividing average expression in cells containing sgNfib/x by sgLacZ (for *Nfib/x* KO) or by dividing cells containing *Nfib*-GFP plasmid by control plasmid (for *Nfib* OE). Rows are annotated by broad cell state and experiment, columns are annotated by gene list. Stars indicate differential expression which was inferred using Seurat’s FindMarker-function with default parameters (two-sided Wilcoxon rank-sum test); *P < 0.01 (Bonferroni-adjusted). ePN, excitatory projection neuron precursor; IN, interneuron precursor; iPN, inhibitory projection neuron precursor. Source data

**Extended Data Fig. 6:**
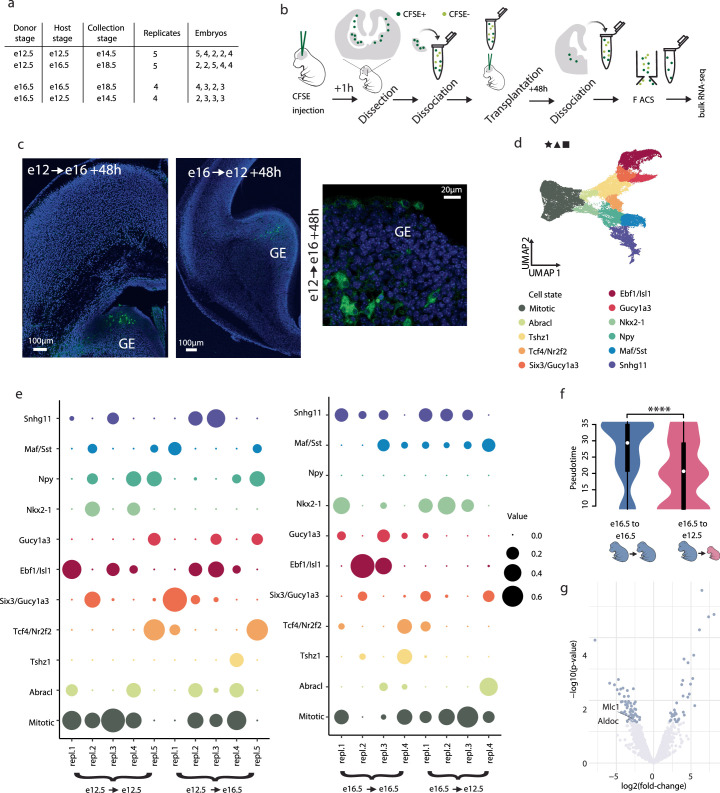
Homo- and hetero-chronic transplantation datasets. **a**, Overview table of homo- and hetero-chronic transplantation experiment datasets: donor, host, and collection stages, number of replicates and number of embryos for each condition. **b**, Schematic representation of the experimental procedure for AP labeling and homo- and heterochronic transplantation. **c**, Images of coronal brain sections after FT^+^ APs transplantation. APs labelled with CFSE; GE, ganglionic eminence (n=4). **d**, UMAP plot of the combined dataset utilized for cluster reference. **e**, Predicted cell state composition in each replicate. **f**, Distribution of transplanted cells along pseudotime in AP_e16.5 → e16.5_ and AP_e16.5 → e12.5_; two-sided Wilcoxon rank sum test (**** adjusted P<0.0001, P = 4.19^*^10^−13^, n=1000). Median is indicated by points, 25th and 75th quantiles are inidcated by hinges. Whiskers show hinges +/- 1.5*inter-quartile range. **g**, Differentially expressed genes between AP_e16.5 → e16.5_ and AP_e16.5 → e12.5_; 1<log2FC<–1, Benjamini-Hochberg adjusted P<0.05 (two-sided Wald test). Only genes downstream of NFIB, MEIS2 and TCF4 are labelled. Source data

To investigate whether the extrinsic environment influences maturation competence in APs at different stages, we conducted homo- and heterochronic transplantation experiments, assessing cell pseudotime scores and expression of genes downstream of NFIB, TCF4 and MEIS2. We injected CFSE into the ventricles of donor mouse embryos at e12.5 and e16.5. At 1 h later, we dissected and dissociated the GE, obtaining a cell suspension that included FT-labeled APs, unlabeled BPs and unlabeled precursor cells. The cell suspension was transplanted homo- and heterochronically into host embryos via intraventricular injection (AP_e12.5 → e12.5_, AP_e12.5 → e16.5_, AP_e16.5 → e16.5_, and AP_e16.5 → e12.5_), as described previously^40^. At 48 h after transplantation, we collected the GE from host embryos, isolated FT^+^ cells by FACS and assessed their transcriptome using bulk RNA-seq (Fig. 4a and Extended Data Fig. 6a,b). By the time of collection, cells had already entered the tissue and begun migrating away from the ventricular zone (Extended Data Fig. 6c).

Using clusters from our combined scRNA-seq data as a reference, we applied Bisque^41^ to estimate the proportions of different neuronal states in the transplantation-derived datasets (Extended Data Fig. 6d,e and Supplementary Fig. 13a). We then assigned a maturation score to each replicate by using the average pseudotime score per reference cluster and weighted it according to the inferred cell state proportions (Methods). The pseudotime scores were higher when APs were transplanted into an e16.5 environment (AP_e12.5 → e16.5_, AP_e16.5 → e16.5_) compared to an e12.5 environment (AP_e12.5 → e12.5_, AP_e16.5 → e12.5_; Fig. 4b and Extended Data Fig. 6f). To identify transcriptomic differences induced by transplantation, we filtered the count matrix by highly variable genes from our combined scRNA-seq datasets and used DeSeq2 (ref. ^42^) for differential expression analysis (Fig. 4c and Extended Data Fig. 6g). Notably, *Nfib* and many of its downstream genes (among other genes) exhibited increased expression in AP_e12.5 → e16.5_ compared to AP_e12.5 → e12.5_. We did not observe significantly downregulated genes (Fig. 4c). Furthermore, only two genes downstream of NFIB (*Mlc1* and *Aldoc*) were significantly downregulated in AP_e16.5 → e12.5_ compared to AP_e16.5 → e16.5_. These findings indicate an involvement of the extrinsic environment in shaping the maturation competence of transplanted cells. The patterns of pseudotime and gene expression were reminiscent of the recipient stage. The gene expression changes observed after transplantation suggest that maturation competence may be associated more closely with the acquisition of specific genes rather than their loss, although this remains to be further explored.

### *Nfib* loss impairs maturation and overexpression promotes maturation

**Extended Data Fig. 7:**
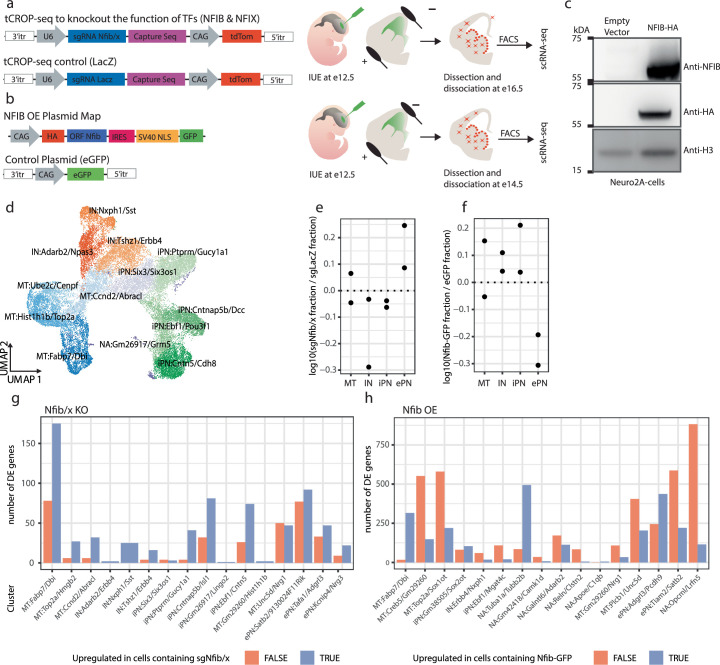
Experimental perturbation of Nfib and associated phenotype. **a**, Design of sgRNAs for tCROP-seq experiments and overview of experimental procedure. **b**, Design of plasmids for *Nfib* OE experiments and overview of experimental procedure. **c**, Western blot showing increased expression of exogenous NFIB in Neuro2A-cells (n=1). **d**, UMAP embedding of inhibitory precursors and their progenitors in *Nfib/x* KO. Cells are colored by clusters, which are annotated based on broad cell states and the top two marker genes; mt: mitotic; IN: interneuron precursor; iPN: inhibitory projection neuron precursor; NA: not assigned. **e**, Proportion change in *Nfib/x* KO. Cells were grouped into broad cell states, by aggregating clusters. **f**, Proportion change in *Nfib* OE. Cells were grouped into broad cell states by aggregating predicted labels. **g**, Number of differentially expressed (DE) genes per cluster between conditions in *Nfib/x* KO. Color indicates positive or negative enrichment. **h**, Number of DE genes per cluster between conditions in *Nfib* OE. Color indicates positive or negative enrichment. Source data

To functionally validate the influence of NFIB on maturation competence, we employed two experimental approaches: in vivo CRISPR perturbation using transposon-based CRISPR droplet sequencing (tCROP-seq)^11^ to knockout *Nfib* and *Nfix* (*Nfib/x*), and overexpression of *Nfib*. For the tCROP-seq experiment, we performed IUE at e12.5 to introduce single-guide RNAs (sgRNAs) and Cas9 vectors targeting progenitor cells into the GE of wild-type mouse embryos (C57BL/6). We used a piggyBac-based IUE strategy at e12.5 for stable delivery, ensuring that the CRISPR-induced perturbation is maintained in progenitors and passed on to both early- and late-born cells, which are captured jointly for transcriptomic analysis. To maximize perturbation efficiency, we employed a combination of sgRNAs targeting both *Nfib* and *Nfix* (sgNfib and sgNfix), as *Nfix* is part of the same downstream transcriptional program through which NFIB coordinates maturation^36^ (Supplementary Figs. 11a and 20) and may compensate for *Nfib* loss. This dual-target approach aimed to ensure robust perturbation of the NFIB pathway. Control embryos were targeted with sgRNAs for LacZ (sgLacZ). Cortices, striata and olfactory bulbs were dissected at e16.5, and cells were enriched by FACS based on tdTomato fluorescence, which labeled sgRNA-expressing cells, and green fluorescent protein (GFP) fluorescence, which labeled Cas9-expressing cells (Methods). To minimize batch effects, we pooled cells from several embryos which received either sgNfib and sgNfix or sgLacZ and then performed multiplexed scRNA-seq (Extended Data Fig. 7a). In total, we acquired four replicates for the *Nfib*/*x* knockout, consisting of two biological replicates, each with two technical replicates.

The *Nfib* overexpression experiments were conducted in a similar manner by targeting progenitor cells in the GE at e12.5 via IUE. A pCAG vector encoding *Nfib*-GFP was used, along with an additional pCAG vector encoding red fluorescent protein (RFP) to facilitate efficient sorting, due to the low GFP signal produced by the *Nfib* overexpression vector. Control embryos were electroporated with the pCAG-eGFP vector. At e14.5, cortices and striata were dissected, and RFP^+^ cells were enriched by FACS for *Nfib* overexpression, while GFP^+^ cells were used as controls (Extended Data Fig. 7b). We acquired two biological replicates for *Nfib* overexpression and control. To confirm the production of functional protein from the exogenous *Nfib* overexpression vector, *Nfib* overexpression was performed in Neuro2A cells (Methods). Detection of NFIB and the hemagglutinin (HA) tag was carried out by western blot using anti-HA and anti-NFIB antibodies (Extended Data Fig. 7c).

The transcriptomic landscape of cells collected from *Nfib/x* knockout and *Nfib* overexpression was profiled using scRNA-seq and analyzed using a standard Seurat pipeline (Methods). For *Nfib/x* knockout, the filtered dataset contained 47,079 cells with 5,887 cells containing sgNfib and/or sgNfix and 30,328 cells containing sgLacZ. Cells were clustered and annotated by their top two marker genes (Fig. 4d and Supplementary Fig. 14a). Our dataset contained a fraction of excitatory precursors expressing the marker genes *Neurod2* and *Neurod6* (Supplementary Fig. 17). This probably reflects that targeting GE progenitors via IUE also labels some progenitors of excitatory neurons, presumably located at the interface of ventral and dorsal progenitor domains^8,11^. For inferring pseudotime scores, we used Monocle 3 on subsetted precursors of inhibitory neurons and their progenitors (Fig. 4e, Extended Data Fig. 7d and Methods).

Cells from *Nfib* overexpression experiments were processed using a workflow similar to *Nfib/x* knockout. To address batch-specific variability, including contributions from ambient RNA observed in one replicate, we excluded cells containing hemoglobin transcripts and performed batch correction using Harmony^43^. The filtered dataset included 30,019 cells, comprising 5,859 *Nfib*-GFP^+^ cells and 7,702 eGFP^+^ control cells. We applied label transfer, using the integrated dorso–ventral scRNA-seq dataset as a reference, labeling cells as ‘not assigned’ when their maximum prediction score was below 0.5, thus minimizing the impact of low-confidence assignments on downstream analyses (Fig. 4f and Supplementary Fig. 14b,d). Pseudotime scores were calculated using Monocle 3 (Fig. 4g).

We aggregated clusters (in *Nfib/x* knockout) or predicted labels (in *Nfib* overexpression) into broad groups consisting of mitotic cells, interneurons, projection neurons and excitatory precursors (Supplementary Fig. 14c,d) and calculated the proportional changes in these cell states following *Nfib/x* knockout or *Nfib* overexpression (Methods). Across both experiments, the relative fraction of mitotic cells remained stable (Extended Data Fig. 7e,f). However, the overall fraction of postmitotic inhibitory neurons decreased with *Nfib/x* knockout and increased with *Nfib* overexpression (Extended Data Fig. 7e,f).

The decrease in inhibitory neuron precursors following *Nfib/x* knockout was not uniform across finer-grain clusters of interneurons and projection neurons, with only some clusters being affected (Fig. 4h,i). To refine our understanding of cell state shifts, we utilized Milo^44^—a computational tool designed to infer differential abundance within neighborhoods of single cells. Milo identified localized changes in population structure, showing decreased abundances of inhibitory precursors in neighborhoods corresponding to clusters of both interneurons and projection neurons (*Adarb2_Npas3*, *Nxph1_Sst*, *Ebf1_Pou3f1*, and *Cntn5_Cdh8*) (Supplementary Fig. 15a,b). This finding was consistent with cell proportion changes observed across clusters (Fig. 4h).

Additionally, we analyzed the effect of the perturbation on postmitotic precursors of excitatory neurons, finding an increased abundance in *Nfib/x* knockout and a decreased abundance in *Nfib* overexpression (Extended Data Fig. 7e,f). Changes in abundance were further explored using Milo for *Nfib/x* knockout, with some cell states being more affected than others. Detailed results are provided in the Supplementary Data (Supplementary Fig. 16a–c).

In addition to changes in cell state proportions, we also observed alterations in gene expression and pseudotime trajectories. To assess the transcriptional impact of *Nfib/x* knockout or *Nfib* overexpression, we performed differential gene expression analyses between conditions within each cluster (Methods) and quantified the number of differentially expressed genes. In *Nfib/x* knockout and *Nfib* overexpression, pronounced changes in cell state abundance were not always accompanied by a high number of differentially expressed genes. For example, APs in both *Nfib/x* knockout and *Nfib* overexpression displayed a relatively high number of differentially expressed genes despite minimal changes in cell proportions (Extended Data Fig. 7g,h). Next, we aimed to determine whether the affected genes were direct targets of NFIB. We overlapped differentially expressed genes from *Nfib/x* knockout or *Nfib* overexpression with genes whose promoters were bound by NFIB in CUT&RUN data (Supplementary Fig. 15c,d). We observed that more than half of the differentially expressed genes were directly bound by NFIB (62.9% for *Nfib/x* knockout and 60.1% for *Nfib* overexpression). The true proportion of direct NFIB targets is probably higher, as genes regulated via enhancer regions were not considered.

To determine maturation shifts along the pseudotime trajectory following perturbation, we compared pseudotime scores across conditions, with APs showing significantly reduced scores in *Nfib/x* knockout and significantly increased scores in *Nfib* overexpression (Wilcoxon rank-sum test) (Fig. 4j). However, this effect did not extend to more mature cell states, as only BPs in the *Nfib* overexpression showed a significant increase in pseudotime scores (Fig. 4j).

Next, we focused on genes with various functional roles during neurogenesis and visualized their aggregated expression differences across conditions in each cluster for both the *Nfib/x* knockout and *Nfib* overexpression experiments (Fig. 4k). A detailed analysis of gene expression changes, including validation using in situ hybridization images from the Allen Brain Institute’s Developing Mouse Brain Atlas^45^ and insights into the regulation of cytoskeleton, progenitor markers, migration genes and markers of postmitotic cell states, is provided in the Supplementary Data (Supplementary Fig. 19).

Together, the shift in pseudotime maturation scores of APs, changes in postmitotic precursor abundance and alterations in gene expression underscore the regulatory influence of NFIB. However, not all postmitotic cell states were affected equally, highlighting a complex, cell state-dependent regulatory landscape.

## Discussion

We describe the regulatory mechanisms that govern progenitor competence during the development of inhibitory neurons. Our results show that the competence of progenitors of GABAergic neurons is tied closely to the timing of neurogenesis. This timing influences primarily the maturation of their neuronal progeny, with little effect on their differentiation. Both cell-intrinsic attributes (including TF expression, chromatin remodeling and reorganization of the gene regulatory network) as well as cell-extrinsic cues collectively define stage-specific maturation competence. The results suggest a mechanism that may compensate for variations in the time available for migration and network integration between early- and late-born neurons. Data presented in this study are accessible through an interactive online platform, enabling users to explore scRNA-seq, scATAC-seq, CUT&RUN and eGRN datasets (https://mayerlab.net/mouse-inhibitory-neuron-development/).

The birthmark of maturation is probably passed from GABAergic mitotic progenitors to their progeny through chromatin priming at regulatory regions. NFIB, a member of the NFI family of TFs, exhibited extensive genomic binding and high regulatory activity at late stages of neurogenesis. NFI TFs are known to regulate both neuronal and glial lineages during central nervous system development^22^. They function as cofactors for FOXP2 to facilitate chromatin opening and activate neuronal maturation genes in human subplate and deep-layer cortical neurons^24^. NFI TFs have been shown to regulate chromatin through various mechanisms, such as binding to chromatin modifiers^46^, opening chromatin^47^ and by influencing the distribution of histone modifications, probably through the establishment of chromatin domain boundaries^48^. Furthermore, NFIX has been shown to regulate the timely generation of intermediate progenitor cells from radial glia, partly through the transcriptional upregulation of *Insc*^49^. In our data, NFIB promotes and forms partnerships with essential regulators of GABAergic interneuron and projection neuron development, such as TCF4 and MEIS2 (refs. ^11,37^), and binds to promoters of the *Dlx* family of genes, known to promote the identity and expansion of GABAergic neurons^50^. We propose that NFIB may prime enhancer regions in APs of the GE, initiating chromatin remodeling and leading to stage-specific maturation competence.

We found that the overexpression of *Nfib* in GE progenitors accelerated the acquisition of postmitotic neuronal identity, whereas knockout of *Nfib* and *Nfix* delayed maturation. Although these findings highlight the regulatory role of NFIB, the mechanisms remain unclear. Knockout studies in mice have shown that deficiency in these genes leads to various neurodevelopmental defects, including hydrocephalus, corpus callosum malformations and enlarged ventricles^51^, while neuronal progenitors in the mouse cortex and retina fail to differentiate^49,52,53^. In humans, haploinsufficiency of *NFI* genes results in overlapping neurodevelopmental phenotypes, including intellectual disability, macrocephaly and brain anomalies^21^.

The decrease in inhibitory neuron precursors observed after *Nfib/x* knockout was not uniform across all interneuron and projection neuron branches (Fig. 4h,i and Supplementary Fig. 15a,b). This suggests that NFIs specifically regulate the maturation of certain GABAergic neuron lineages, rather than affecting all inhibitory neuron subtypes uniformly.

Other mechanisms, such as the rate of metabolic activity in mitochondria^54^ or selective translation of epigenetic modifiers^55^, have been proposed to govern neuronal maturation. The release of epigenetic barriers sets the timing of maturation in neural progenitor cells, with key factors including EZH2, EHMT1/2 and DOT1L^56,57^. In our study, we observed that *Ezh2*—a member of the Polycomb repressive complex 2 (PRC2)—is depleted in APs following *Nfib/x* knockout in inhibitory neurons. Interestingly, in *Nfib*-knockout mice, *Ezh2* showed upregulated expression within hippocampus and neocortex^58^. Together, this suggests an interaction between NFIB and members of PRC2, albeit following different regulatory rules in the GE and neocortex.

The maturation shift may involve an interplay of extrinsic and intrinsic factors, as GABAergic progenitors in heterochronic transplantation adjust to the host environment by acquiring altered gene expression patterns. Potential extrinsic contributors include feedback from newborn cells^59^, extracellular vesicle exchange^60^ and tissue stiffness^61^.

Although several studies described temporal and spatial differentiation patterns in GABAergic neurons^62^, there is little evidence of a fate birthmark transmitted from APs to their daughter cells. By contrast, glutamatergic neurons display a birthdate-dependent generation of transcriptomically distinct postmitotic cells that is linked to a progression in the differentiation competence of their progenitors^3–5,63^. Although a sequential mechanism does not appear to drive diversity in the GE, several other factors have been implicated in the generation of distinct GABAergic neuron types. These include the mode of cell division^64,65^, cell-cycle length^66–68^, progenitor heterogeneity^69^, TFs that transduce patterning signals^11,70–76^ and spatially restricted enhancer activation^11^. This study contributes to the broader discourse on neuronal maturation, offering insights into the plasticity and commitment of GABAergic progenitors.

## Methods

### Animals

All experiments were conducted according to institutional guidelines of the Max Planck Society and the regulations of the local government ethical committee (Beratende Ethikkommission nach §15 Tierschutzgesetz, Regierung von Oberbayern). All procedures were approved by the Bavarian government for the Max Planck Institute for Biological Intelligence (ROB-55.2-2532.Vet_02-18-81 and ROB-55.2-2532.Vet_02-23-87). All mouse colonies were maintained in accordance with protocols approved by the Bavarian government. Mice were group housed in isolated ventilated cages (room temperature 22 ± 1 °C, relative humidity 55 ± 5%) under a 12-h/12-h dark/light cycle with ad libitum access to food and water. Mouse strains used are the following: wild-type C57BL/6NRj, Tg(dlx6a-cre)1Mekk (Dlx6-Cre; JAX:008199)^10^, Rosa26LSL-tdTomato (Ai9; JAX:007909)^77^, Tg(Nes-flpo/ERT2)1Alj (Nes-FlpoER; MGI:5532191)^78^, *Gad2*^*tm1(cre/ERT2)Zjh*^ (Gad2-CreER, JAX:010702)^79^ and Ai65(RCFL-tdT)-D (Ai65D, JAX:021875)^80^. Embryos were staged in days postcoitus, with e0.5 defined as 12:00 of the day a vaginal plug was detected after overnight mating.

### Cell line

Mouse Neuro2A neuroblastoma cells (ECACC, cat. no. 89121404) were cultured in Dulbecco’s modified Eagle medium (DMEM, Sigma, cat. no. D6429) supplemented with 10% (v/v) fetal bovine serum (FBS, Sigma, cat. no. F9665) and containing 1% (v/v) antibiotics (100 U ml^−1^ penicillin, 100 mg ml^−1^ streptomycin, Sigma, cat. no. P0781). Neuro2A cells were incubated at 37 °C in a 5% CO_2_ humidified atmosphere and passaged twice a week. Cell passage numbers were limited to no more than ten.

### scRNA-seq datasets: sample and library preparation

Three to six brains from Dlx5/6-Cre::tdTomato mouse embryos were collected at e12.5, e14.5 or e16.5 in ice-cold L-15 medium containing 5% FBS. GEs were dissected manually and dissociated with the Miltenyi Bio Tech Neural Tissue Dissociation Kit (P) (cat. no. 130-092-628) on a gentleMACS Dissociator according to the manufacturer’s protocol. From the same brains, cortical and striatal regions were dissected, dissociated and FACS-enriched for tdTomato-positive cells using a SY3200 Cell Sorter (software WinList3D, v.8.0.2) or BD FACSAria III Cell Sorter (BD FACSDiva Software, v.8.0.2) with a 100 μm nozzle. tdTomato-positive neurons from the cortex and striatum were pooled with neurons from the GEs, and scRNA-seq was performed. For experiments using the 10x Genomics platform, Chromium Single Cell 3′ Library and Gel Bead Kit v.3 (cat. no. PN-1000075), Chromium Single Cell 3′ Chip Kit v.3 (cat. no. PN-1000073) and Chromium i7 Multiplex Kit (cat. no. PN-120262) were used according to the manufacturer’s instructions. Additionally, Chromium Single Cell 3′ Library and Gel Bead Kit v.3.1 (cat. no. PN-1000268), Chromium Single Cell 3′ Chip Kit v.3.1 (cat. no. PN-1000127) and Dual Index Kit TT Set A (cat. no. PN-1000215) were used according to the manufacturer’s instructions in the Chromium Single Cell 3′ Reagents Kits v.3.1 User Guide (Dual Index). Libraries were quantified using a BioAnalyzer (Agilent) and sequenced either on an Illumina NextSeq500 or Novaseq at the Genomics Core Facility of the Helmholtz Center, at the Next-Generation Facility of the Max Planck Institute of Biochemistry or at MLL Münchner Leukämielabor GmbH.

### TrackerSeq datasets: sample and library preparation

Timed pregnant mice were anesthetized with isoflurane (5% induction, 3% during the surgery) and treated with the analgesic Metamizol (WDT). IUE of the TrackerSeq library was performed at e16.5 as previously described^8^. Embryos were injected unilaterally in the lateral ventricle with 700 nl of DNA plasmid solution made of 0.5 μg μl^−1^ pEF1a-pBase (piggyBac transposase) and the TrackerSeq library 0.5 μg μl^−1^, diluted in endo-free TE buffer and 0.002% Fast Green FCF (Sigma). Embryos were then electroporated with five electric pulses (50 V, 50 ms at 1 Hz) with a square-wave electroporator (BTX, ECM830). The transcriptome libraries were prepared utilizing the 10x Genomics platform as described previously. The lineage barcode library retrieved from RNA was amplified with a standard NEB protocol for Q5 Hot Start High-Fidelity 2× Master Mix (cat. no. M094S) in a 50-μl reaction, using 10 μl of cDNA as a template. Specifically, each PCR contained the following: 25 μl Q5 High-fidelity 2× Master Mix, 2.5 μl 10 μmol P7 indexed reverse primer, 2.5 μl 10 μmol i5 indexed forward primer, 10 μl molecular-grade H_2_O and 10 μl cDNA. The PCR protocol for amplifying TrackerSeq lineage libraries was: (1) 98 °C for 30 s, (2) 98 °C for 10 s, (3) 63 °C for 20 s, (4) 72 °C for 10 s, (5) repeat steps 2–4 for 11–18 times, (6) 72 °C for 2 min and (7) 4 °C hold. Libraries were purified with a dual-sided SPRI selection using Beckman Coulter Agencourt RNAClean XP beads (Beckman Coulter, cat. no. A63987) and quantified with a BioAnalyzer.

### FT transcriptome datasets: sample and library preparation

Timed pregnant mice were anesthetised with isoflurane and treated with the analgesic Metamizol as previously described. A CFSE working solution was prepared by adding 8 μl of dimethylsulfoxide and 1 μl of Fast Green to one vial of CellTrace CFSE (CellTrace CFSE, Life Technologies, cat. no. C34554) for a final concentration of 10 mM, following the instructions described previously^9^. For FT_e12.5+6h_ and FT_e16.5+6h_, 500 nl of CFSE working solution was injected into ventricles of wild-type C57BL/6NRj embryos at e12.5 and e16.5, respectively. The abdominal wall was then closed, and the embryos were left to develop until collection. After 6 h, GEs were dissected manually and dissociated on the gentleMACS Dissociator according to the manufacturer’s protocol. FT positive cells with high intensity (>10^5^) were sorted using FACS (Supplementary Fig. 1g) and scRNA-seq was performed. For FT_e12.5+96h_, 500 nl of CFSE working solution was injected into ventricles of Dlx5/6-Cre::tdTomato embryos at e12.5. After 96 h, the striatum and cortex were dissected and dissociated on the gentleMACS Dissociator. FT and tdTomato-positive cells were sorted using FACS, and scRNA-seq was performed.

### FT chromatin accessibility datasets: sample and library preparation

Sample preparation followed the same protocol described for FT_e12.5+6h_ and FT_e16.5+6h_ in the previous sections. Single-cell ATAC-seq was performed according to the Chromium Single Cell ATAC Reagent Kits v.1 user guide (10x Genomics). FACS-sorted cells were centrifuged at 500*g* for 5 min at 4 °C and resuspended in 100 μl chilled diluted lysis buffer and incubated for 5 min at 4 °C; 1 ml of chilled wash buffer was added to the lysed cells and mixed five times with a pipette, followed by centrifugation at 500*g* for 5 min at 4°C. The isolated nuclei were counted (using a c-chip hemocytometer) and resuspended in an appropriate volume of chilled diluted nuclei buffer to reach the desired final nuclei concentration. The nuclei were used immediately to generate single-cell ATAC libraries, followed by paired-end sequencing on the Illumina NextSeq500 platform.

### Electrophysiological analysis of membrane potential in progenitors

After decapitation, the brain was placed in an ice-cold cutting solution saturated with a mixture of 95% O_2_ and 5% CO_2_ containing: 30 mM NaCl, 4.5 mM KCl, 1 mM MgCl_2_, 26 mM NaHCO_3_, 1.2 mM NaH_2_PO_4_, 10 mM glucose and 194 mM sucrose. The brain was cut at a thickness of 350 μm on a vibratome (Leica VT1000S), and the slices were transferred into an artificial cerebrospinal fluid solution containing: 124 mM NaCl, 4.5 mM KCl, 1 mM MgCl_2_, 26 mM NaHCO_3_, 1.2 mM NaH_2_PO_4_, 10 mM glucose and 2 mM CaCl_2_ (310–320 mOsm), saturated with 95% O_2_ and 5% CO_2_ at approximately 32 °C for 1 h before being moved to room temperature. Finally, the brain slices were transferred to a recording chamber continuously perfused with artificial cerebrospinal fluid solution saturated with 95% O_2_ and 5% CO_2_ at 30 °C to 32 °C. Patch pipettes were prepared from filament-containing borosilicate micropipettes (World Precision Instruments) using a P-1000 micropipette puller (Sutter Instruments), with a resistance of 10–12 MΩ. The intracellular solution contained 130 mM potassium gluconate, 10 mM KCl, 2 mM MgCl_2_, 10 mM HEPES, 2 mM Na-ATP, 0.2 mM Na_2_GTP, pH 7.35, and 290 mOsm. Slices were visualized with a fluorescence microscope equipped with IR–DIC optics (Olympus BX51). Data were obtained using a MultiClamp 700B amplifier, Digidata 1550 digitizer (Molecular Devices) and the software Clampex v.10.3 (Molecular Devices). Data were sampled at 10 kHz, filtered at 2 kHz and analyzed with Clampfit (Molecular Devices). For resting membrane potential recordings, when stable, the membrane potential was recorded for 2 min and the average obtained every 30 s was used.

### RNAscope on FT-labeled cells

FT labeling of the cells was performed as for FT_e12.5+6h_ and FT_e16.5+6h_, by injecting CFSE in the mouse brain ventricles at e12.5 and e16.5 and collecting 6 h later. The brains were fixed overnight in 4% paraformaldehyde solution in 1× PBS at 4 °C. After two washes with 1× PBS, the brains were treated in a series of sucrose solutions (10%, 20% and 30%) for 12 h each. The brains were then embedded in optimal cutting temperature compound. Coronal slices of 10 μm thickness were obtained using a cryostat (Leica CM 3050), placed on Superfrost Plus slides, and washed three times with 1× PBS to remove optimal cutting temperature compound residues. Sample pretreatment and hybridization steps were executed according to the manufacturer protocol (RNAscope Multiplex Fluorescent Reagent Kit v.2, cat. no. 323100 from Advanced Cell Diagnostics). Akoya Biosciences Opal fluorophores 570 (1:1,500) and 690 (1:5,000) and Bio-Techne RNAscope Probes for Ascl1 (cat. no. 313291) and Gad2 (cat. no. 439371) were utilized for signal detection. The slides were mounted with Prolong Gold Antifade Mountant (P10144 from Invitrogen), stored in the dark at room temperature overnight and visualized using a Zeiss AxioScan Z.1.

### Transplantation datasets: sample and library preparation

To generate the AP_e12.5 → e12.5_, AP_e12.5 → e16.5_, AP_e16.5 → e16.5_ and AP_e16.5 → e12.5_ datasets, timed pregnant mice were anesthetised with isoflurane and treated with the analgesic Metamizol as described previously. To target APs, injection of CFSE working solution was performed into wild-type C57BL/6NRj embryos at e12.5 and e16.5. After 1 h, FT^+^ APs were collected from three to six embryonic brains. After manual dissection of the GEs in ice-cold L-15 medium containing 5% FBS, the tissue was dissociated on a gentleMACS dissociator according to the manufacturer’s protocol. Cells were resuspended in ice-cold HBSS containing 10 mmol EGTA and 0.1% Fast Green to a final concentration of 40,000 cells μl^−1^ to 80,000 cells μl^−1^. The cell suspension was split into two separate pools, and 1 μl was injected homo- or heterochronically into the ventricles of embryonic brains at e12.5 or e16.5. After 48 h, GEs were dissected and dissociated as described above. CFSE-labeled cells were isolated with flow cytometry and centrifuged at 23*g* for 5 min at 4 °C. Total RNA-seq libraries were prepared using the SMART-Seq Stranded Kit (cat. no. 634442, Takara), according to standard manufacturer’s protocol (low-input workflow; PCR 1, five cycles and PCR 2, 12–15 cycles). The library quality was assessed by using a Qubit Flex Fluorometer (cat. no. Q33327, ThermoFisher Scientific) and a 4200 TapeStation (cat. no. G2991BA, Agilent). A total of ten samples were multiplexed and sequenced in a lane of a NovaSeq 6000 SP flow cell with the 100 cycles kit for paired-end sequencing (2 × 60 bp) to reduce sequencing batch effects (100 pM final loading, 42 M reads per sample on average). BCL raw data were converted to FASTQ data and demultiplexed by the bcl2fastq Conversion Software (Illumina).

### Cut&Run sample and library prep

CUT&RUN was performed using the EpiCypher CUTANA CUT&RUN protocol. Two biological replicates were included for each antibody condition: anti-NFIB, anti-H3K4me3 and anti-IgG. GEs were manually dissected from the brains of C57BL/6N mouse embryos collected at e16.5 in ice-cold L-15 medium supplemented with 5% FBS. Tissue dissociation was performed using the Neural Tissue Dissociation Kit (P) (Miltenyi Biotec, cat. no. 130-092-628) on a gentleMACS Dissociator, following the manufacturer’s protocol. For each sample, 750,000 cells were processed with the following antibodies: anti-NFIB (Sigma, cat. no. HPA003956), anti-H3K4me3 (EpiCypher, cat. no. 13-0041) and anti-IgG (EpiCypher, cat. no. 13-0042), according to manufacturer’s protocols. Library preparation was carried out using the NEB Next Ultra II DNA Library Prep Kit for Illumina (New England Biolabs, cat. no. E7645).

### *Nfib/x* tCROP-seq sample and library preparation

#### gRNA selection and vector construction

The sgRNAs were designed using CRISPick for CRISPRko^81,82^ and validated with inDelphi^83^ for high frame shift efficiency. At least three sgRNAs per gene were cloned into the backbone using ssDNAs oligo (IDT) and NEBuilder HiFi DNA Assembly (NEB, cat. no. E5520). The backbone was a piggyBac plasmid (Addgene, cat. no. 229995), which encodes tdTomato and sgRNA under the human U6 promoter and has a capture sequence at the scaffold of sgRNA for 10x feature barcode retrieval (cs1 incorporated at the 3′ end^84^. The efficiency of the sgRNAs was measured in Neuro2A cells. Cells were transfected with pCAG-Cas9-eGFP (gift from R. Platt) and sgRNA plasmids using FuGENE 6 Transfection Reagent (Promega, cat. no. E2691). After 48 h, cells were sorted with Beckman Coulter Cytoflex SRT for tdTomato and eGFP. Genomic DNA was extracted using the Quick-DNA Miniprep Plus Kit (Zymo, cat. no. D4068), and the region around the sgRNA target was amplified using Q5 polymerase (NEB, cat. no. M094S) with primers listed in Supplementary Table 2, and subsequently sent to Microsynth Seqlab GmbH for Sanger sequencing. Knockout efficiency was quantified using TIDE software^85^. The results for selected sgRNAs are shown in Supplementary Table 2.

#### Mice and in utero surgeries

C57BL/6NRj wild-type females (from inhouse breeding) were crossed to wild-type males. Embryos were staged in days postcoitus, with E0.5 defined as 12:00 of a day that a vaginal plug was detected after overnight mating. Timed pregnant mice were anesthetized with isoflurane (5% induction, 2.5% during surgery) and treated with the analgesic Metamizol (WDT). A microsyringe pump (Nanoject III Programmable Nano-liter Injector, DRUM3-000-207) was used to inject 700 nl of DNA plasmid solution made of 0.6 μl of pEF1a-pBase (piggyBac transposase) and pCAG-Cas9-eGFP (both a gift from R. Platt); and the sgRNA plasmid 0.5–8 μl, diluted in sterile 0.9% NaCl solution and 0.002% Fast Green FCF (Sigma, cat. no. F7252) into the lateral ventricle. Embryos were then electroporated by holding the head between platinum-plated tweezer electrodes (5 mm in diameter, BTX, cat. no. 45-0489) across the uterine wall, while five electric pulses (35 V, 50 ms at 1 Hz) were delivered with a square-wave electroporator (BTX, cat. no. ECM830)^86^. We used these relatively large electrodes to target all areas of the GE (MGE, CGE and LGE). Before preparing brain tissue for scRNA-seq, each brain was examined under a stereo microscope and only brains that met the following criteria were processed for scRNA-seq: (1) dispersed tdTomato-positive neurons throughout the neocortex, (2) dense tdTomato-positive neurons throughout the striatum and (3) tdTomato-positive neurons in the olfactory bulb.

#### Sample collection and sequencing

We collected electroporated brains from mouse embryos at E16.5 in ice-cold Leibovitz’s L-15 Medium (ThermoFisher, cat. no. 11415064) with 5% FBS (Sigma, cat. no. F9665). The same medium was used during flow cytometry sorting. Papain dissociation system (Worthington, cat. no. LK003150) was carried out according to the protocol described previously^87^ on the gentleMACS Octo Dissociator (Miltenyi Biotec). To isolate positive cells for tdTomato and eGFP, flow cytometry was done using a Beckman Coulter Cytoflex SRT with a 100-μm nozzle. After sorting, 16,000 individual cells per sample, in PBS (Lonza) with 0.02% BSA (ThermoFisher), were loaded onto a 10x Genomics Chromium platform for Gel Beads-in-emulsion (GEM) and cDNA generation carrying cell- and transcript-specific barcode using the Chromium Single Cell 3′ Reagent Kit v.3.1 with Feature Barcoding technology (cat. no. PN-1000121) following manufacture protocol (document number CG000205, 10x Genomics). We generated 3′ gene expression and sgRNA libraries according to the manufacturer’s manual using the Chromium Library v.3.1 kit (cat. no. PN-1000121), Feature Barcode Library Kit (cat. no. PN-1000079) and Single Index Kit (cat. no. PN-1000213) from 10x Genomics. The quantification of the libraries was performed with the 4200 TapeStation.

### *Nfib* overexpression sample and library preparation

#### Mice and in utero surgeries

Timed pregnant mice were anesthetised with isoflurane and treated with the analgesic Metamizol as previously described. IUE was performed at e12.5. Embryos were injected unilaterally in the lateral ventricles with 700 nl of DNA plasmid solution. For the *Nfib* overexpression samples, the plasmids used were pCAGG-NFIB2 (Addgene, cat. no. 112700) and pBCAG-mRFP (Addgene, cat. no. 40996). The target concentrations for each embryo were 1.5 μg of pCAGG-NFIB2, 1 μg of pBCAG-mRFP and 0.1% Fast Green to aid injections. For control embryos, the plasmid pBCAG-eGFP (Addgene, cat. no. 40973) was used at a concentration of 1 μg with 0.1% Fast Green. The abdominal wall was then closed, and the embryos were left to develop until collection.

#### Sample collection and library preparation

At e14.5, electroporated brains were collected in ice-cold Leibovitz’s L-15 Medium with 5% FBS. Cell were dissociated on a gentleMACS Dissociator according to the manufacturer’s protocol. For *Nfib* overexpression samples, RFP-positive cells were isolated using FACS, while eGFP-positive cells were sorted for control samples. Cells were collected in PBS supplemented with 1% BSA. Libraries were prepared using the Chromium Next GEM Single Cell 3′ Reagent Kit v.3.1 (10x Genomics), according to the manufacturer’s instructions. Quality control of the libraries was performed using TapeStation and qubit to ensure proper fragment distribution and concentration. Sequencing was carried out on an Element AVITI sequencer.

### Western blotting

Neuro2A cells (2 × 10^6^ cells per well) were seeded in 10-cm dishes the day before transfection. The following day, cells were transfected with 8 μg of *Nfib*-GFP or of empty pcDNA plasmid using Turbofect transfection reagent (cat. no. R0533, ThermoFisher). Cells were collected 72 h after transfection by scraping in ice-cold PBS and centrifuging at 400*g* for 5 min at 4 °C. Nuclei were extracted by suspending in 2 ml of buffer A (10 mM HEPES (pH 7.9), 10 mM KCl, 10 mM EDTA, 0.5% Igepal, 1 mM DTT and complete protease inhibitor (Roche, cat. no. 4693132001) and incubating on ice for 10 min with vortexing at maximum speed every 2 min for 10 s. Nuclei were then collected by centrifugation (800*g*, 10 min, 4 °C) and the supernatant was removed carefully. Nuclei were disrupted in 0.15 ml lysis buffer (50 mM HEPES (pH 7.5), 150 mM NaCl, 5 mM EGTA, 1.5 mM MgCl_2_, 1% Triton X-100, 1% glycerol and complete protease inhibitor) by shaking at 1,500 rpm in a Thermomixer for 2 h at 2 °C, with vortexing at maximum speed for 10 s every half hour. Samples were centrifuged (16,000*g*, 15 min, 4 °C) and the supernatant collected. Cell lysates were diluted to 1× in 4× NuPAGE LDS sample buffer (ThermoFisher, cat. no. NP0007) with NuPAGE sample reducing agent (ThermoFisher, cat. no. NP0009). Samples were then boiled for 7 min at 90 °C, 25 μl of each sample was loaded onto NuPAGE Bis-Tris Mini Protein Gels, 4–12% (cat. no. NP0322) for electrophoresis and transferred to polyvinylidene fluoride membrane (ThermoFisher, cat. no. PB5210) using a Power-Blotter Semi-dry transfer system (ThermoFisher Scientific). Membranes were blocked with 5% milk, and then incubated in blocking buffer with rabbit anti-NFIB (1:1,500, Atlas Antibodies, cat. no. HPA003956), or anti-HA (Proteintech, cat. no. 51064-2-AP, 1:5,000) and Anti-H3K4me3 (1:10,000, Sigma, cat. no. H0164) overnight at 4 °C. Proteins were detected using horseradish peroxidase-labeled secondary anti-rabbit antibodies (Thermo Scientific, cat. no. G21234) and developed using SuperSignal West Pico PLUS Chemiluminescent Substrate (Thermo Scientific, cat. no. 34577).

### scRNA-seq TrackerSeq and FT transcriptome datasets: preprocessing and merging

Sequencing reads were processed using Cell Ranger v.3.0.2 or v.6.1.2 (ref. ^88^), using the mouse reference genome mm10 v.2.1.0. Resulting count matrices were analyzed using the Seurat package v.4.3.0 (ref. ^89^) in R v.4.1.0. For each dataset, high-quality cells were filtered by the number of genes and mitochondrial read fraction (Extended Data Fig. 1b). Subsequently, counts were normalized and corrected for sequencing depth using Seurat’s NormalizeData function. Cell-cycle assignments for each cell were calculated using the cell-cycle gene list from ref. ^90^. After identification of highly variable features as described in ref. ^91^, we calculated scaled gene expression values by applying *z*-normalization to the 2,000 most variable genes, while simultaneously regressing out unwanted sources of variation: number of counts per cell, number of genes per cell, mitochondrial read fraction and estimated difference between cell-cycle phases (ScaleData function). The FindClusters function with default parameters was used to identify cell clusters. The FindAllMarkers function was used to identify cluster marker genes. Clusters with marker genes of excitatory neurons (for example, *Neurod1*, *Neurod6*, *Tbr2*) or non-neuronal cells (for example, *Apoe*, *Olig1*, *Flt1*, *Pdgfra*) were filtered out and excluded from the following steps. Raw counts of samples from scRNA-seq, TrackerSeq and FT datasets were merged using the Seurat package and aligned using Monocle3 v.1.0.0 (ref. ^13^). For this purpose, the scaled matrix from the Seurat object was converted into a Monocle3 object of the cell_data_set class and preprocessed without the default normalization as the dataset was already normalized. Batch correction was performed using Batchelor v.1.8.1 (ref. ^92^) followed by Leiden clustering (using fine resolution) and dimensional reduction using UMAP. A developmental trajectory was fitted as a principal graph through fine clusters based on the UMAP embedding. The root of the trajectory was defined as the cells with the highest *Nes* gene expression, identified in the ‘Fabp7’ cluster. A pseudotime score was assigned to each cell based on its projected position on the trajectory. Leiden clustering (using coarser resolution in Monocle 3) identified distinct clusters of cell states. Marker genes specific to each cluster were identified by running differential expression analysis (in Seurat) using the FindAllMarkers function. Clusters were aggregated into broad cell states according to marker gene expression of cell states: *Nes* and *Fabp7* for AP; *Ascl1* and *Ccnd2* for BP; *Tcf4*, *Lhx6* and *Sst* for interneurons; and *Meis2*, *Ebf1* and *Isl1* for projection neurons. Clusters were annotated manually based on broad cell state and marker gene expression. The transition between mitotic and postmitotic cells was defined by selecting the highest pseudotime score of mitotic clusters as the threshold.

To relate postmitotic precursors to mature cell types in the adult brain, we performed label transfer on cells in branch tips using scRNA-seq data of GABAergic neuron populations at P10 from ref. ^8^ as a reference dataset. For label transfer we used code from ref. ^6^, which does a correlation-based mapping of cells with the possibility to not assign a label if prediction scores are low.

### scRNA-seq datasets and published datasets: analysis

We downloaded raw counts of e13.5 and e15.5 datasets from ref. ^8^ (GSE IDs: GSM5684874, GSM5684875, GSM5684876, GSM5684877, GSM5684878 and GSM5684879) and raw counts from the developing mouse somatosensory cortex^3^ at stages e12.5 to e16.5 (GSE IDs: GSM4635073, GSM4635074, GSM4635075, GSM4635076 and GSM4635077). The count matrices were merged with our scRNA-seq datasets to create a combined Seurat object. We filtered cells based on mitochondrial read fraction (≤10%). Normalization, scaling, batch correction, dimensionality reduction and clustering were performed as previously described. Clusters were manually annotated based on top marker gene expression. For cells originating from ref. ^3^, we utilized the annotations available from the original paper^3^.

Relative fraction of cells per cell state were calculated for each stage and tissue origin (dorsal versus ventral) separately, by counting the number of cells per cell state and normalizing by the total number of cells. For cells from the dorsal telencephalon we used annotation from ref. ^3^ for defining cell states. For cells from the ventral telencephalon we used transcriptomic clusters. To ensure that results were not biased by different methods for defining cell states, we repeated our analysis based on cell types defined on fine clusters.

We screened for genes that are variable along the pseudotime in inhibitory and excitatory lineages with the following steps: (1) for each lineage, we binned cells from each stage into ten sections based on their inferred pseudotime. (2) Enriched genes were selected based on two criteria: high expression and high gene abundance. High expression was inferred by calculating the fold change between the expression in all cells inside the bin compared to all cells outside the bin. High gene abundance was calculated by comparing the fraction of cells that express a gene inside versus outside the bin (a gene was considered to be expressed in a cell if its scaled expression value was higher than 0.5). (3) A normal distribution was fitted to the changes in expression and abundance. Significantly enriched genes were selected when the difference in expression and abundance was higher than the corresponding average difference plus two times the standard deviation of the corresponding fitted distribution. (4) Steps two and three were repeated for each bin. (5) The trajectory of inhibitory neurons diverged as cells leave the cell cycle; therefore, we ran this algorithm for each branch independently and only considered genes that appeared in at least two out of five branches. (6) Finally, by taking the union of dynamic genes of all stages in inhibitory or excitatory lineages, we created a stable set of genes that are dynamic along pseudotime, but conserved across stages.

To compare the correlation of apical progenitors between excitatory and inhibitory datasets, we selected the inhibitory datasets from our study (e12.5, e14.5, e16.5) and the corresponding timepoints from the excitatory datasets. Apical progenitor cells were then subset from these datasets, followed by normalization and scaling. During this process, we regressed out the effects of mitochondrial genes, as well as the number of genes and gene counts per cell. Two thousand highly variable genes for apical progenitors were identified using the Seurat function FindVariableFeatures. Next, the average expression of the identified genes was calculated for each cluster: excitatory e12.5, e14.5, e16.5 and inhibitory e12.5, e14.5, e16.5. Pearson’s correlation analysis was performed based on these genes. To assess the robustness of the results, we downsampled the datasets to ensure comparable unique molecular identifier counts across all datasets (nCount_RNA < 10,000). Despite this adjustment, similar correlation patterns were observed. Marker genes for excitatory (e12.5, e14.5 and e16.5) and inhibitory (e12.5, e14.5 and e16.5) apical progenitors were identified using the FindAllMarkers function in Seurat (min.pct = 0.25, logfc.threshold = 0.25). The intersection between these marker genes and highly variable genes revealed that 30% of the highly variable genes were also marker genes.

### TrackerSeq datasets: analysis

TrackerSeq barcode reads were preprocessed as described previously^8^. To assess the clonal coupling between cell states, we calculated *z*-scores between clusters^93^. The *z*-score is defined as the number of shared barcodes relative to randomized data, with values ranging from positive (coupled clusters) to negative (anticoupled clusters). We utilized these random permutations to calculate empirical *P* values. For coupled pairs of clusters, the null hypothesis is that the observed coupling is not higher than random coupling. Conversely, for anticoupled pairs, the null hypothesis is that the observed coupling is not lower than random couplings. A random coupling is in contradiction to the null hypothesis when a permutation for one pair of clusters scores above the observed coupling (for positively coupled pairs), or below the observed coupling (for negatively coupled pairs). The relative fraction is reflected in the empirical *P* value, which was consequently corrected for multiple comparisons using the Benjamini–Hochberg (false discovery rate) method^94^.

Clones were identified as ‘dispersing’ or ‘nondispersing’ depending on whether their cells were distributed in several or just one branch tip, respectively. We tested whether the transcriptome of mitotic progenitors in ‘nondispersing’ clones was predictive of their postmitotic state. ‘Nondispersing’ clones were grouped by their postmitotic cell state in both TrackerSeq_e12.5+96h_ and TrackerSeq_e16.5+96h_ combined. Separate data frames were created for mitotic and postmitotic subsets of each group. Pearson correlation coefficients were calculated between pairs of gene expression within different subsets, generating all the possible combinations of pairs within the columns of the data frames. The subsets of postmitotic clones, mitotic clones, and randomly selected mitotic cells were all correlated to the postmitotic reference group.

### FT transcriptome datasets: analysis

FT datasets were subset from the common trajectory, and differences in pseudotime between cohorts were assessed using a two-sided Wilcoxon rank-sum test (conf.level = 0.95). Next, differential gene expression was calculated between the postmitotic fractions of FT_e12.5+6h_ and FT_e16.5+6h_. The expression of these genes was visualized in a heatmap for FT_e12.5+6h_, FT_e16.5+6h_ and FT_e12.5+96h_.

For the Venn diagram, marker genes for each cohort (FT_e12.5+6h_, FT_e16.5+6h_ and FT_e12.5+96h_) were calculated separately with the FindMarkers function in Seurat. Genes with average log_2_FC > 0.25 and adjusted *P* value < 0.05 were then intersected, to find common markers across the cohorts.

### FT chromatin datasets: analysis

The raw sequencing data (BCL files) were converted to the fastq format using Cellranger-atac mkfastq function from Cell Ranger ATAC v.1.2.0 (ref. ^95^). The reads were aligned to the mm10 (GRCm38) mouse reference genome and fragment files were generated using the Cellranger-atac count function. Both timepoints included two replicates and the aligned fragment files were converted to arrow files and analyzed further using the ArchR package v.1.0.1 (ref. ^18^). Dimensionality reduction was performed using latent semantic indexing, followed by batch correction using Harmony v.0.1.1 (ref. ^43^). To extract the trajectories of interest and integrate them into an ArchR project, we employed the getTrajectory and addTrajectory functions, respectively. To visualize enriched motifs, we generated pseudotime heatmaps.

### FT chromatin datasets: temporal dynamics and coverage plots

Peak calling was performed using the addReproduciblePeakSet function, which runs MACS2 (ref. ^96^) to identify marker peaks for FT_e12.5+6h_ and FT_e16.5+6h_ datasets. Peaks were classified by either identifying for peaks that overlap across stages, based on genomic position (e12.5-enriched, e16.5-enriched or nonenriched peaks); or by conducting differential peak analysis with *P* value cutoff *P* = 0.05 using ArchR getMarkerFeatures function. As an additional quality check, we counted the number of reads that map to peak regions and calculated their fraction in respect to all reads (Supplementary Fig. 9e). For both scATAC-seq and H3K4me3 ChIP–seq datasets, we calculated peak coverage for each peak category using the ScoreMatrixList function.

### FT chromatin datasets: TF footprint analysis

Footprint analysis was carried out on FT_e12.5+6h_ and FT_e16.5+6h_ datasets using TF occupancy prediction tool: TOBIAS v.0.14.0 (ref. ^25^). We employed the Jaspar nonredundant motif database^97^ as the primary reference source for motif data. Bias correction was performed to generate corrected bigwig files using the ATACorrect function with default parameters. Footprint scores were calculated on corrected bigwig files using the FootprintScores function and differential binding TFs were detected using BINDetect function. Predicted TFs were categorized as significant based on two criteria: their differential binding score (greater than 0.2 for e12.5 and less than −0.4 for e16.5: referred to as change) and the −log_10_ of the *P* value from the statistical test against a background model. The footprints were visualized using the PlotAggregate and PlotHeatmap functions.

### FT chromatin datasets: cobinding analysis

To detect co-occurring TF binding sites, we utilized TF-COMB v.1.1 (ref. ^38^). A distinct CombObj was created by loading unique peak sets (e12.5 and e16.5) identified previously. TF binding sites were identified within the peak regions followed by market basket analysis. TFs co-occurring with NFIB were then subsetted and further assessed for their cobinding (cosine score) and binding events via dot plot.

### FT transcriptome and chromatin datasets: gene regulatory network prediction

We used SCENIC+ v.0.1 (ref. ^26^) to predict GRNs for CFSE-labeled cells at e12.5 and e16.5. As scRNA-seq and scATAC-seq data were unpaired, we created a common annotation, by defining broad cell states (AP, BP and precursor) in the transcriptomic data, merging clusters based on marker gene expression. Annotations in the scATAC-seq data were created by applying label transfer, based on gene-scores predicted by ArchR. These broad cell states were split by stage, resulting in six stage-specific cell states (Supplementary Fig. 10a). SCENIC+ performs co-accessibility analysis of regions and links regions to upstream TFs by searching for enriched TF motifs in regions. To make this analysis more coherent with previous results, we used the previously calculated peak set from ArchR as input for SCENIC+, instead of recalculating a new peak set using pycisTopic^98^. Following the Scenic+ workflow, we created topics of coaccessible regions and performed binarization and motif enrichment of regions in the 20 most important topics. Networks were created by aggregating ten cells from both modalities of corresponding cell states into pseudocells and then inferring TFs and regions that are predictive of a gene, based on co-accessibility and motif enrichment. The regions considered for a gene have to lie within a genomic interval of 150 kb up- and downstream of the gene. The results are ‘eRegulons,’ that is, regulatory triplets of one TF, bound regions and corresponding target genes. For each eRegulon, the activity in each cell was calculated using area under the curve (AUC)-scores^27^. Each eRegulon was filtered using standard filtering (apply_std_filtering_to_eRegulons function) and high-quality eRegulons were selected by filtering for eRegulons where TF expression and AUC scores correlated more than 0.5 or less than −0.5.

We reconstructed cell-state-specific subnetworks by running AUC-binarization (binarize_AUC-function). Here, we filtered for (1) eRegulons that are active in at least 50% of cells within a corresponding cell state and (2) corresponding target genes that have a higher normalized expression than 0.5 (normalized expression is log transformed after correcting for sequencing depth). Cell-state-specific networks (APs, BPs and precursors) were created by merging the corresponding e12.5- and e16.5-subnetwork using the igraph library v.1.5.0 (ref. ^99^). Stage-specific networks (e12.5 and e16.5) are similarly created by merging subnetworks of APs, BPs and precursors of the same stage. In both approaches, the merged networks consisted of the union of vertices and edges. Gene ontology enrichment analysis of target genes was performed using DAVID with default parameters^100^.

### CUT&RUN preprocessing and analysis

Raw sequencing reads were mapped to the *Mus musculus* reference genome (mm10) using Bowtie2 (ref. ^101^) using parameters –end-to-end –very-sensitive –no-mixed –no-discordant –phred33 -I 10 -X 700 for mapping of inserts 10–700 bp in length. Reads were also aligned to the spike-in genome. Following alignment, duplicates were marked using Picard (https://broadinstitute.github.io/picard/). Peaks were called using MACS2 (*P* value cutoff of 1 × 10^−4^) using IgG as background. Signal tracks were then generated in bigWig format for visualization in genome browsers. Peak heatmaps and genome browser profiles were generated by using fluff heatmap and fluff profile function^102^. Enriched motifs were identified using findMotifsGenome.pl of HOMER^39^. Motif heatmap was generated using visualization package^103^. Peak heatmap signal quantification was done using normalized read counts (reads per kilobase per million mapped reads) by averaging read coverage from peak summit ±500 bp.

### Transplantation datasets: analysis

We used the Galaxy web platform on the public server at usegalaxy.eu to analyze the data^104^. Paired-end reads were trimmed with the Trimmomatic tool and quality control was performed with FastQC. Reads were mapped to the mouse reference genome using the HISAT2 algorithm^105^ and the number of reads per annotated genes was counted using featureCounts^106^. After fragments per kilobase of transcript per million mapped reads normalization of the count matrices, the proportion of single-cell states within each replicate was inferred with Bisque v.1.0.5 (ref. ^41^) by using the annotated combined single-cell clusters as reference. A weighted pseudotime score was assigned to each replicate by calculating the median of the pseudotime score per cluster from the combined single-cell datasets. For differential gene expression analysis, the count matrices were subset by variable genes of inhibitory neuron datasets and DESeq2 v.1.42.0 was used^42^.

### tCROP-seq datasets: analysis

Reads from transcriptome and single-guide libraries of all four replicates were mapped to the mm10 reference genome and demultiplexed using Cell Ranger v.8.0.1 (ref. ^88^). Single-cell count matrices from transcriptomic libraries of the four replicates were merged in Seurat^89^. We excluded cells with more than 10% fraction of mitochondrial reads, cells predicted to be doublets according to DoubletFinder^107^ and cells that contained both sgNfib/sgNfix and sgLacZ. After cleaning the dataset, count data were log-normalized and variable features were calculated using Seurat’s FindVariableFeatures function. Log-normalized expression data of variable genes was scaled using Seurat’s ScaleData function, while regressing out effects of read-depth (number of genes and number of unique molecular identifiers) and fraction of mitochondrial reads. Based on the scaled expression matrix, we calculated low-dimensional representations of cells (principal component analysis and UMAP). Cells were clustered using FindNeighbors and FindClusters functions with default parameters. Clusters were annotated by calculating marker genes for each cluster, using FindAllMarkers function, and naming clusters by their top two positively enriched marker genes.

By counting the number of cells that contain either sgNfib and/or sgNfix or contain sgLacZ in each cluster per biological replicate, we calculated proportion changes induced by *Nfib/x* knockout in each cluster. Proportion change was calculated by dividing the number of cells that contained sgNfib/x per cluster by the number of cells that contained sgLacZ per cluster and applying log_10_ transformation to the result of the division. This was repeated for broad cell states, which were generated by aggregating individual clusters. To infer the effect of perturbation on gene expression we performed DE-analysis between cells containing sgNfib/x and cells containing sgLacZ in each cluster using Seurat’s FindMarkers function with default parameters. The number of differentially expressed genes per cluster was inferred by setting a cutoff of the adjusted *P* value being smaller than 0.01.

Owing to spread of CRISPR constructs during IUE in the ventricle, progenitors of excitatory neurons that lie dorsal of GEs were also targeted. Based on expression of marker genes for excitatory precursors (*Eomes*, *Neurod2*, *Neurod6*) and transcriptomic clustering, we filtered the dataset for inhibitory precursors and their progenitors, by removing cells belonging to five clusters (*Gm29260_Hist1h1b*, *Unc5d_Nrg1*, *Satb2_9130024F11Rik*, *Tafa1_Adgrl3* and *Kcnip4_Nrg3*). Processing of the inhibitory subset, from inferring variable features to clustering, was repeated in the same way as described above. We inferred pseudotime scores for the inhibitory subset by converting scaled data and UMAP representation into a cell–dataset object. This was used to infer a trajectory of single cells and infer pseudotime scores using Monocle 3 (ref. ^92^). We ran Milo^44^ on both inhibitory and excitatory subsets after excluding cells that did not contain any guide RNA. Seurat preprocessing was repeated and Milo was executed by setting *k* = 40 for both subsets of data.

### *Nfib* overexpression datasets: analysis

For mapping reads from *Nfib* overexpression experiments we added the sequence of *Nfib*-GFP, eGFP and RFP to the mm10 reference genome, using Cellranger’s mkref function. Subsequently, mapping and demultiplexing were performed for all four experiments using Cellranger (v.8.0.1) with the custom reference genome. Single-cell count matrices from all four experiments were merged in Seurat. We detected high levels of ambient RNA (as indicated by ambient expression of hemoglobin genes). Therefore, cells that expressed both *Hbb-bt* and *Hbb-bs* were removed from further analysis. Additionally, we removed cells with more than 20% of mitochondrial reads and cells that were predicted to be doublets according to DoubletFinder^107^. As described above, read counts were normalized, variable features were inferred, data were scaled and we performed principal component analysis. To account for differences in data quality between experiments, we performed batch correction using Harmony^43^. Based on the Harmony-corrected data we inferred clusters and UMAP embedding. Clusters were annotated by their top two marker genes. Assigning clusters to cell states was less straightforward in this dataset, as some clusters contained marker gene expression for several cell states. To circumvent this problem, we ran label transfer using our integrated dorsal–ventral scRNA-seq dataset as a reference. Cells with a low prediction score (<0.5) were labeled as ‘not assigned.’

Proportion changes of predicted cell states upon overexpression of *Nfib*, were calculated by comparing the number of cells that express *Nfib*-GFP (and not eGFP) to the number of cells that express only eGFP per predicted cell state. This was done twice, once for predicted clusters and once for aggregated cell states following the same rationale as for *Nfib/x* knockout. Differentially expressed genes across conditions were inferred by running Seurat’s FindMarkers function for each predicted cluster. Pseudotime scores were inferred in the same way as for tCROP experiments.

### Shiny-based webserver

Results from scRNA-seq experiments (dorsal and ventral wild-type scRNA-seq datasets with FT and TrackerSeq datasets), together with results from scATAC-seq, NFIB CUT&RUN and eGRN analysis were made publicly available via Shiny-based webserver (v.1.9.1)^108^.

### Statistics and reproducibility

The number of replicates and animals used for each dataset is reported as an overview table (Extended Data Fig. 1a). Analyses were carried out using R v.4.1 or Python v.3. Transcriptome and chromatin accessibility studies were performed with the same bioinformatic pipeline between conditions. The sample size was chosen empirically or based on preliminary data to provide a sufficient level of statistical power for detecting indicated biological effects. No statistical methods were used to predetermine sample sizes, but our sample sizes are similar to those reported in previous publications^8^. No data were excluded from the analyses. The experiments were not randomized. Statistical tests used for each analysis are specified in the corresponding figure legends.

### Reporting summary

Further information on research design is available in the Nature Portfolio Reporting Summary linked to this article.

## Online content

Any methods, additional references, Nature Portfolio reporting summaries, source data, extended data, supplementary information, acknowledgements, peer review information; details of author contributions and competing interests; and statements of data and code availability are available at 10.1038/s41593-025-01999-y.

## Supplementary information

Supplementary Information Supplementary results, discussion and Figs. 1–22.

Reporting Summary

Supplementary Table Table 1. Functional summary of e16.5 enriched genes differentially expressed between FT_e12.5+6h_ and FT_e16.5+6h_. Table 2. Selected sgRNAs list with primers used to analyze CRISPR interference efficiency.

## Source data

Source Data Fig. 1 Correlation coefficients between apical progenitors at different stages; membrane potential of progenitors in the GE and cortex and observed interaction sizes between postmitotic cell states in TrackerSeq datasets.

Source Data Fig. 2 Abundance of cell states in FT cohorts; shift in pseudotime across FT cohorts; differentially expressed genes between postmitotic cells; marker gene expression across FT cohorts; aggregated accessibility across stage-enriched sites; differential binding analysis across cohorts.

Source Data Fig. 3 Gene regulatory networks for APs and factors of interest; Enriched motifs in NFIB CUT&RUN peaks.

Source Data Fig. 4 Pseudotime shift in transplanted cells; differentially expressed genes in transplantation experiments; proportion changes of cell states in *Nfib/x* KO and *Nfib* OE experiments; pseudotime shifts in broad cell states in *Nfib/x* KO and *Nfib* OE; changes in expression of factors of interest.

Source Data Extended Data Fig. 1 Quality metrics for scRNA-seq datasets.

Source Data Extended Data Fig. 2 Expression of marker genes.

Source Data Extended Data Fig. 3 Number of mitotic and post-mitotic cells per FT cohort; marker genes of cell states and FT cohorts

Source Data Extended Data Fig. 4 Quantification of peak types; differentially accessible peaks across cohorts; aggregated accessibility patterns.

Source Data Extended Data Fig. 5 Subnetworks for APs, BPs and precursors; combinatorial binding scores; stage-specific subnetworks; TFs upstream of maturation genes.

Source Data Extended Data Fig. 6 Cell state proportions in transplantation experiments; pseudotime shift and differentially expressed genes for e16.5-transplantation experiments.

Source Data Extended Data Fig. 7 Proportion changes across broad cell states in *Nfib/x* KO and *Nfib* OE; number of differentially expressed genes per cell state in *Nfib/x* KO and *Nfib* OE.

Source Data Images for Extended Data Figs. 1, 6 and 7.

## Data Availability

The datasets used in this research article can be downloaded from the Gene Expression Omnibus database. scRNA-seq: GEO accession number GSE255455. scATAC-seq: GEO accession number GSE255104. Total RNA-seq: GEO accession number GSE255103. CUT&RUN: GEO accession number GSE285727. Publicly available datasets used in this study: (1) Encode forebrain H3K4me1 ChIP–seq (GSE82528 and GSE82464); (2) scRNA-seq of somatosensory cortex from ref. ^3^ (GSM4635073, GSM4635074, GSM4635075, GSM4635076 and GSM4635077); (3) scRNA-seq of GE from ref. ^8^ (GSM5684874, GSM5684875, GSM5684876, GSM5684877, GSM5684878 and GSM5684879). As a reference genome we used mm10 (GRCm38): NCBI RefSeq assembly: GCF_000001635.20. In situ hybridization data was accessed from https://developingmouse.brain-map.org/. We developed an interactive web resource for exploring these datasets and comparing findings from the GE with dorsal cortical neurogenesis. The tool can be accessed at: https://mayerlab.net/mouse-inhibitory-neuron-development/. Source data are provided with this paper.

## Code availability

The code to reproduce the data analysis is available via GitHub at https://github.com/mayer-lab/Bright-et-al-2025/tree/main and via Zenodo at 10.5281/zenodo.15195253 (ref. ^109^).
