# Supplementary material for: Temporal control of progenitor competence shapes maturation in GABAergic neuron development in mice
Source: Nat Neurosci. 2025 Jul 8;28(8):1663–75. doi: 10.1038/s41593-025-01999-y (PMC12321585; doi:10.1038/s41593-025-01999-y)
Supplement: Supplementary file 2 — Reporting Summary [file 41593_2025_1999_MOESM2_ESM.pdf]

nature portfolio

Corresponding author(s): Christian Mayer

Last updated by author(s): Apr 1, 2025

Reporting Summary

Nature Portfolio wishes to improve the reproducibility of the work that we publish. This form provides structure for consistency and transparency in reporting. For further information on Nature Portfolio policies, see our Editorial Policies and the Editorial Policy Checklist.

Statistics

For all statistical analyses, confirm that the following items are present in the figure legend, table legend, main text, or Methods section.

n/a	Confirmed	
☐	☒	The exact sample size (*n*) for each experimental group/condition, given as a discrete number and unit of measurement
☐	☒	A statement on whether measurements were taken from distinct samples or whether the same sample was measured repeatedly
☐	☒	The statistical test(s) used AND whether they are one- or two-sided<br>*Only common tests should be described solely by name; describe more complex techniques in the Methods section.*
☒	☐	A description of all covariates tested
☐	☒	A description of any assumptions or corrections, such as tests of normality and adjustment for multiple comparisons
☐	☒	A full description of the statistical parameters including central tendency (e.g. means) or other basic estimates (e.g. regression coefficient) AND variation (e.g. standard deviation) or associated estimates of uncertainty (e.g. confidence intervals)
☐	☒	For null hypothesis testing, the test statistic (e.g. *F*, *t*, *r*) with confidence intervals, effect sizes, degrees of freedom and *P* value noted<br>*Give P values as exact values whenever suitable.*
☒	☐	For Bayesian analysis, information on the choice of priors and Markov chain Monte Carlo settings
☒	☐	For hierarchical and complex designs, identification of the appropriate level for tests and full reporting of outcomes
☐	☒	Estimates of effect sizes (e.g. Cohen's *d*, Pearson's *r*), indicating how they were calculated

Our web collection on statistics for biologists contains articles on many of the points above.

Software and code

Policy information about availability of computer code

Data collection	Fluorescence Activated Cell Sorting: on SY3200 Cell Sorter (software WinList3D version 8.0.2) or BD FACSAria III Cell Sorter (BD FACSDiva Software, version 8.0.2)
Data analysis	The following software/packages were utilized in the manuscript: 10x Genomics Cell Ranger (v3.0.2 or v6.1.2 or v8.0.1), R (v4.1.0), Python (v3.6), Seurat (v4.3.0), Monocle3 (v1.0.0), Batchelor (v1.8.1), Cell Ranger ATAC (v1.2.0), ArchR (v1.0.1), Harmony (v0.1.1), TOBIAS (v0.14.0), TF-COMB (v1.1), SCENICPLUS (v0.1), igraph (v1.5.0), Bisque (v1.0.5), DESeq2 (v1.42.0), Galaxy web platform (v24.2.4.dev0), Clampex (v10.3), DoubletFinder (v2.0.3), Bowtie2 (v2.4.2), MACS2 (v2.2.8), fluff (v3.0.4), David (v2023q4), TIDE (v3.3.0), HOMER (v5.1), miloR (v1.0.0)<br>Link to GitHub: https://github.com/mayer-lab/Bright-et-al-2025/tree/main

For manuscripts utilizing custom algorithms or software that are central to the research but not yet described in published literature, software must be made available to editors and reviewers. We strongly encourage code deposition in a community repository (e.g. GitHub). See the Nature Portfolio guidelines for submitting code & software for further information.

Data

Policy information about availability of data

All manuscripts must include a data availability statement. This statement should provide the following information, where applicable:

- Accession codes, unique identifiers, or web links for publicly available datasets
- A description of any restrictions on data availability
- For clinical datasets or third party data, please ensure that the statement adheres to our policy

The sequencing datasets generated for the current study are available in the Gene Expression Omnibus (GEO) under accession numbers: GSE255455, GSE255104, GSE255103 and GSE285727 . Publicly available gene expression data used for cluster annotation can be accessed as follows: DropViz (dropviz.org) and Allen Brain Atlas (https://portal.brain-map.org/atlases-and-data/bkp/abc-atlas).
Publicly available datasets used are: 1) Encode forebrain H3K4me1 ChIP-seq : GSE82528 and GSE82464 2) Somatosensory cortex scRNA-seq data from Di Bella et al. 2021 GEO: GSM4635073, GSM4635074, GSM4635075, GSM4635076, and GSM4635077; 3) Ganglionic eminences scRNA-seq data from Bandler et al.2022 GEO:GSM5684874, GSM5684875, GSM5684876,GSM5684877, GSM5684878, and GSM5684879.
Reference genome: mm10 (GRCm38): NCBI RefSeq assembly: GCF_000001635.20
ISH data from: https://developingmouse.brain-map.org/

Research involving human participants, their data, or biological material

Policy information about studies with human participants or human data. See also policy information about sex, gender (identity/presentation), and sexual orientation and race, ethnicity and racism.

Reporting on sex and gender	No human research participants in the study.
Reporting on race, ethnicity, or other socially relevant groupings	No human research participants in the study.
Population characteristics	No human research participants in the study.
Recruitment	No human research participants in the study.
Ethics oversight	No human research participants in the study.

Note that full information on the approval of the study protocol must also be provided in the manuscript.

Field-specific reporting

Please select the one below that is the best fit for your research. If you are not sure, read the appropriate sections before making your selection.

☒ Life sciences ☐ Behavioural & social sciences ☐ Ecological, evolutionary & environmental sciences

For a reference copy of the document with all sections, see nature.com/documents/nr-reporting-summary-flat.pdf

Life sciences study design

All studies must disclose on these points even when the disclosure is negative.

Sample size	No methods were used to calculate the sample sizes. The sample size was chosen based on previous publications in the field (Bandler et al. 2022; Govindan et al. 2018; Dvoretskova et al. 2024).
Data exclusions	Clusters with marker genes of excitatory neurons or non-neuronal cells were filtered out and not utilized in the next analysis steps if applicable.
Replication	Single-cell transcriptome: 19 samples including 69 embryos. Single-cell chromatin: 4 samples including 18 embryos. Bulk transcriptome: 18 samples including 44 embryos. CUT&RUN: 3 samples including 9 embryos. All generated replicates were included.
Randomization	Not relevant to this study; no need to prevent selection bias or bias in treatment assignments. Experiments contained negative controls (e.g. transplantation), which control for possible bias.
Blinding	Not relevant to this study; no need to prevent selection bias or bias in treatment assignments.

Reporting for specific materials, systems and methods

We require information from authors about some types of materials, experimental systems and methods used in many studies. Here, indicate whether each material, system or method listed is relevant to your study. If you are not sure if a list item applies to your research, read the appropriate section before selecting a response.

Materials & experimental systems

n/a	Involved in the study	
☐	☒	Antibodies
☐	☒	Eukaryotic cell lines
☒	☐	Palaeontology and archaeology
☐	☒	Animals and other organisms
☒	☐	Clinical data
☒	☐	Dual use research of concern
☒	☐	Plants

Methods

n/a	Involved in the study	
☒	☐	ChIP-seq
☒	☐	Flow cytometry
☒	☐	MRI-based neuroimaging

Antibodies

Antibodies used	Anti-NFIB (Sigma, HPA003956), Anti-H3K4me3 (EpiCypher,#13-0041), Anti-IgG (EpiCypher, #13-0042), Anti-HA (Proteintech, 51064-2-AP)
Validation	Anti-NFIB was previously used in immunohistochemistry experiments in the following publications: Lenk et al., 2022, Clin Pharmacol Ther.; Bunt et al., 2017, Brain Neurosci Adv.<br>Anti-H3K4me3 and Anti-IgG were validated for CUT&RUN experiments by the manufacturer. Anti-HA was validated by the manufacturer.

Eukaryotic cell lines

Policy information about cell lines and Sex and Gender in Research

Cell line source(s)	Mouse Neuro2a neuroblastoma cells (ECACC, 89121404)
Authentication	The cell line was not authenticated.
Mycoplasma contamination	The cell line was not tested for mycoplasma.
Commonly misidentified lines (See ICLAC register)	We did not use misidentified cell lines.

Animals and other research organisms

Policy information about studies involving animals; ARRIVE guidelines recommended for reporting animal research, and Sex and Gender in Research

Laboratory animals	Adult mice were used for breeding, and their embryos at e12.5, e14.5 and e16.5 and pups P08 for brain tissue collection. Mouse strains used are the following: wild type C57BL/6NRj, Tg(dlx6a-cre)1Mekk (Dlx6-Cre), JAX:008199 (Monory et al. 2006), Rosa26LSL-tdTomato (Ai9), JAX::007909 (Madisen et al. 2010), Tg(Nes-flpo/ERT2)1Alj (Nes-FlpoER), MGI:5532191 (Lao et al. 2012), Gad2<tm1(cre/ERT2)Zjh> (Gad2-CreER), JAX:010702 (Taniguchi et al. 2011), Ai65(RCFL-tdT)-D (Ai65D), JAX:021875 (Madisen et al. 2015) were used.
Wild animals	No wild animals were used in this study.
Reporting on sex	The sex was not considered in the study.
Field-collected samples	No field-collected samples were used in this study.
Ethics oversight	Animal Protocol: ROB-55.2-2532.Vet_02-18-81 and ROB-55.2-2532.Vet_02-23-87 from Bavarian government for the Max Planck Institute for Biological Intelligence.All experiments were conducted according to institutional guidelines of the Max Planck Society and the regulations of the local government ethical committee (Beratende Ethikkommission nach §15 Tierschutzgesetz, Regierung von Oberbayern). All mouse colonies were maintained in accordance with protocols approved by the Bavarian government.

Note that full information on the approval of the study protocol must also be provided in the manuscript.

Plants

Seed stocks	No seed stocks were used in this study.
Novel plant genotypes	No novel plant genotypes were used in this study.
Authentication	Not applicable.
