# Supplementary figures and images for: Temporal control of progenitor competence shapes maturation in GABAergic neuron development in mice
Source: Nat Neurosci. 2025 Jul 8;28(8):1663–75. doi: 10.1038/s41593-025-01999-y (PMC12321585; doi:10.1038/s41593-025-01999-y)

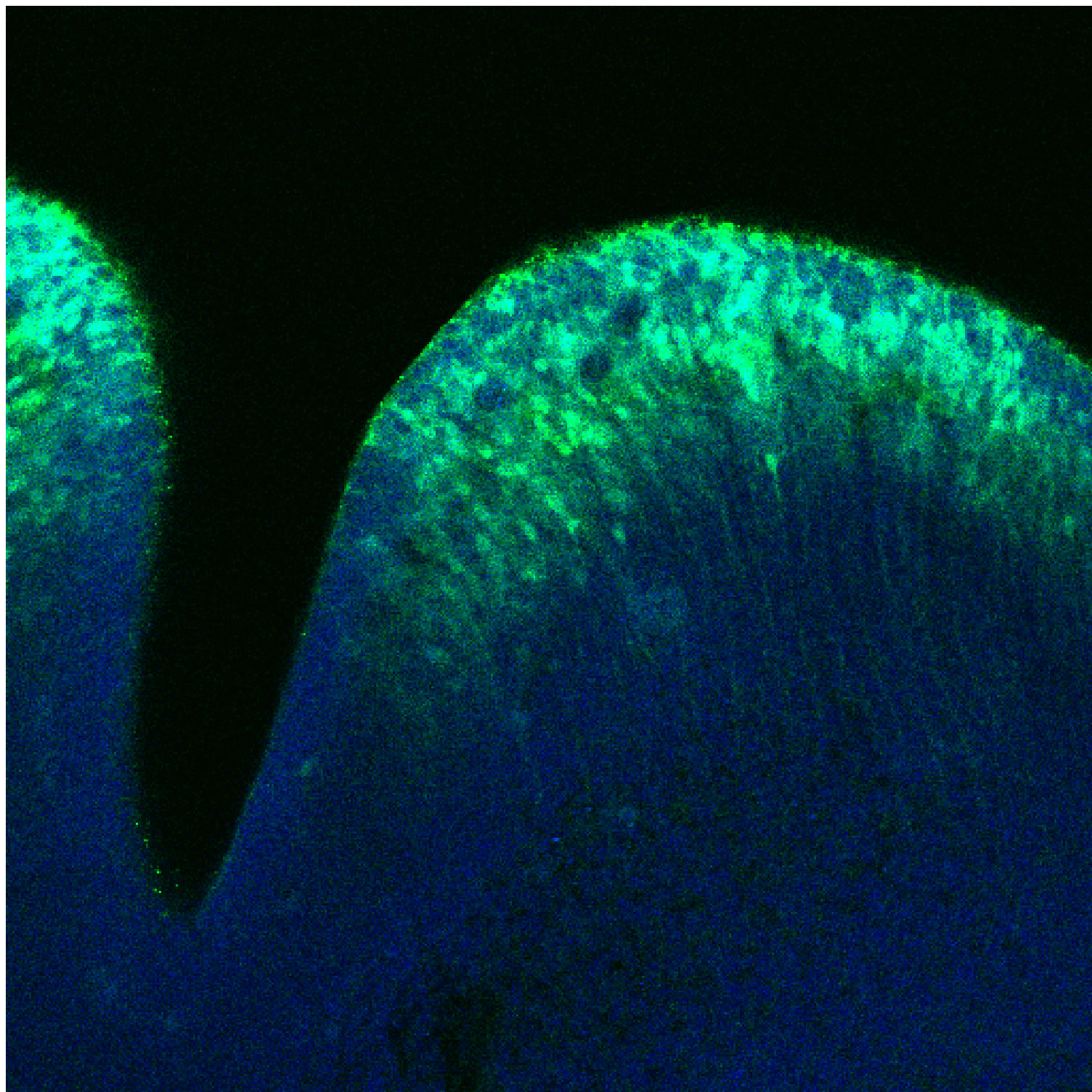

Supplement: Supplementary file 15 — Images for Extended Data Figs. 1, 6 and 7. [file 41593_2025_1999_MOESM15_ESM.zip › image_source/Image_source_ED_Fig1f_e12.5_6h.pdf]

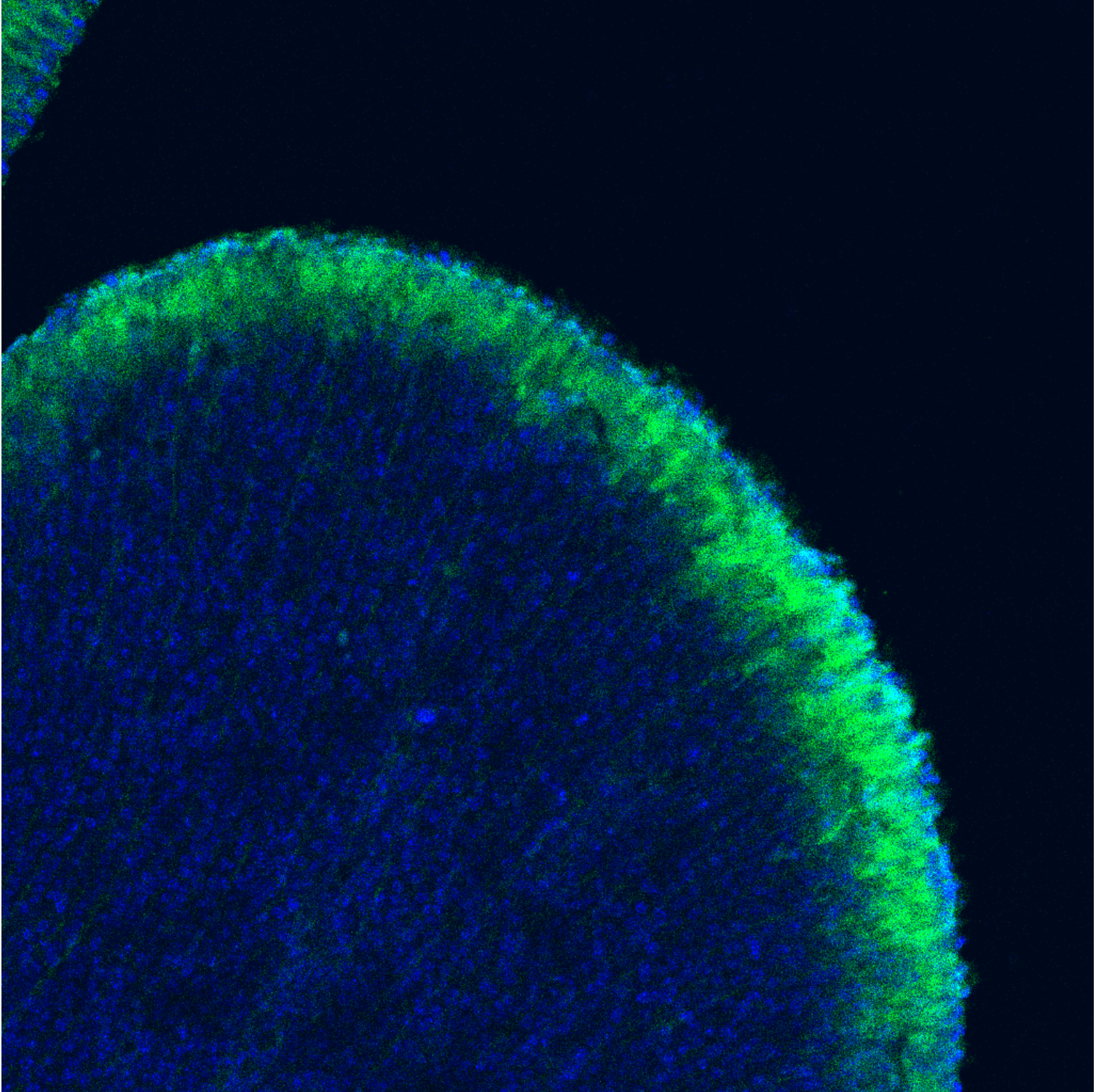

Supplement: Supplementary file 15 — Images for Extended Data Figs. 1, 6 and 7. [file 41593_2025_1999_MOESM15_ESM.zip › image_source/Image_source_ED_Fig1f_e16.5_6h.pdf]

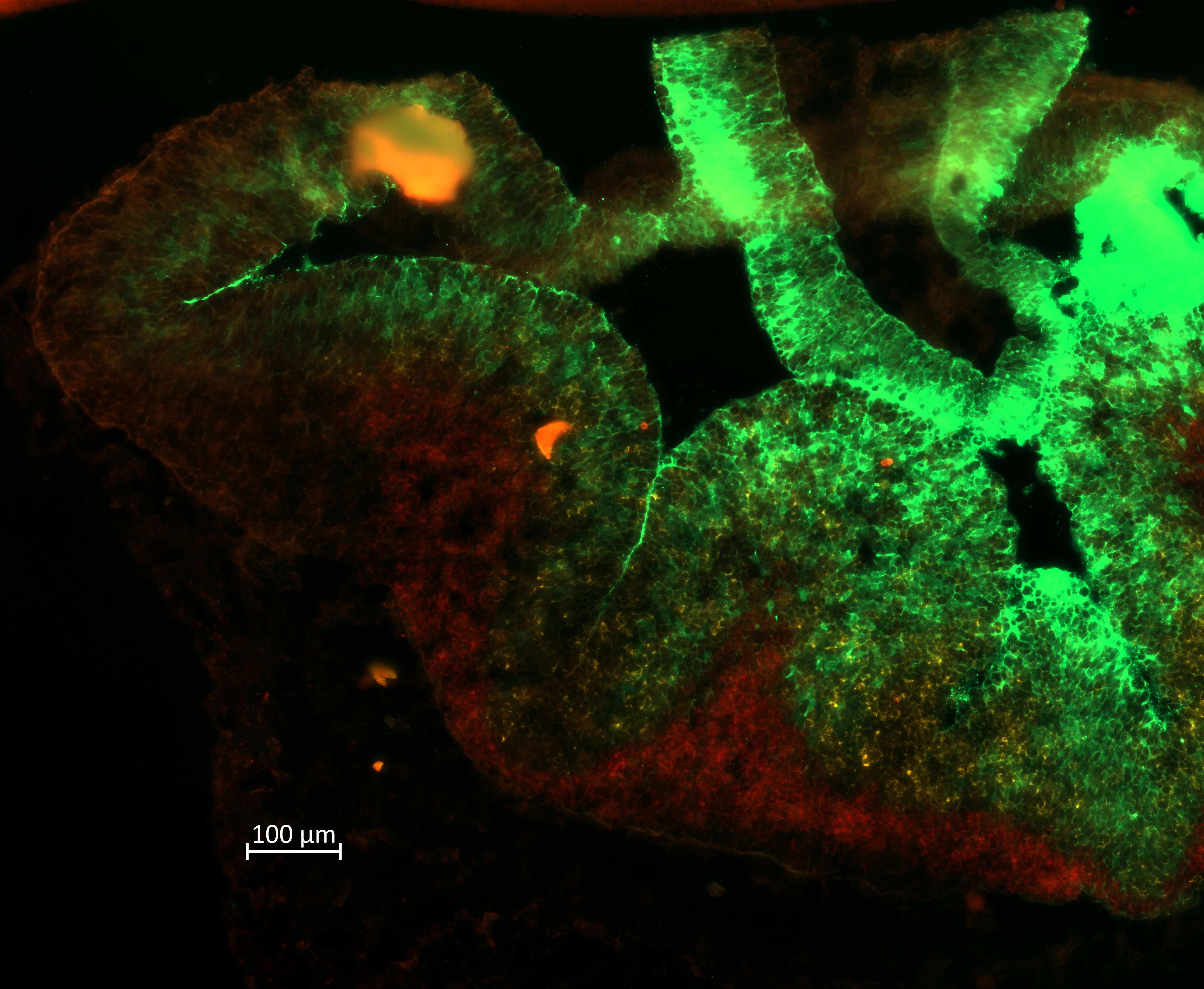

Supplement: Supplementary file 15 — Images for Extended Data Figs. 1, 6 and 7. [file 41593_2025_1999_MOESM15_ESM.zip › image_source/Image_source_ED_Fig1h_Part1.tif]

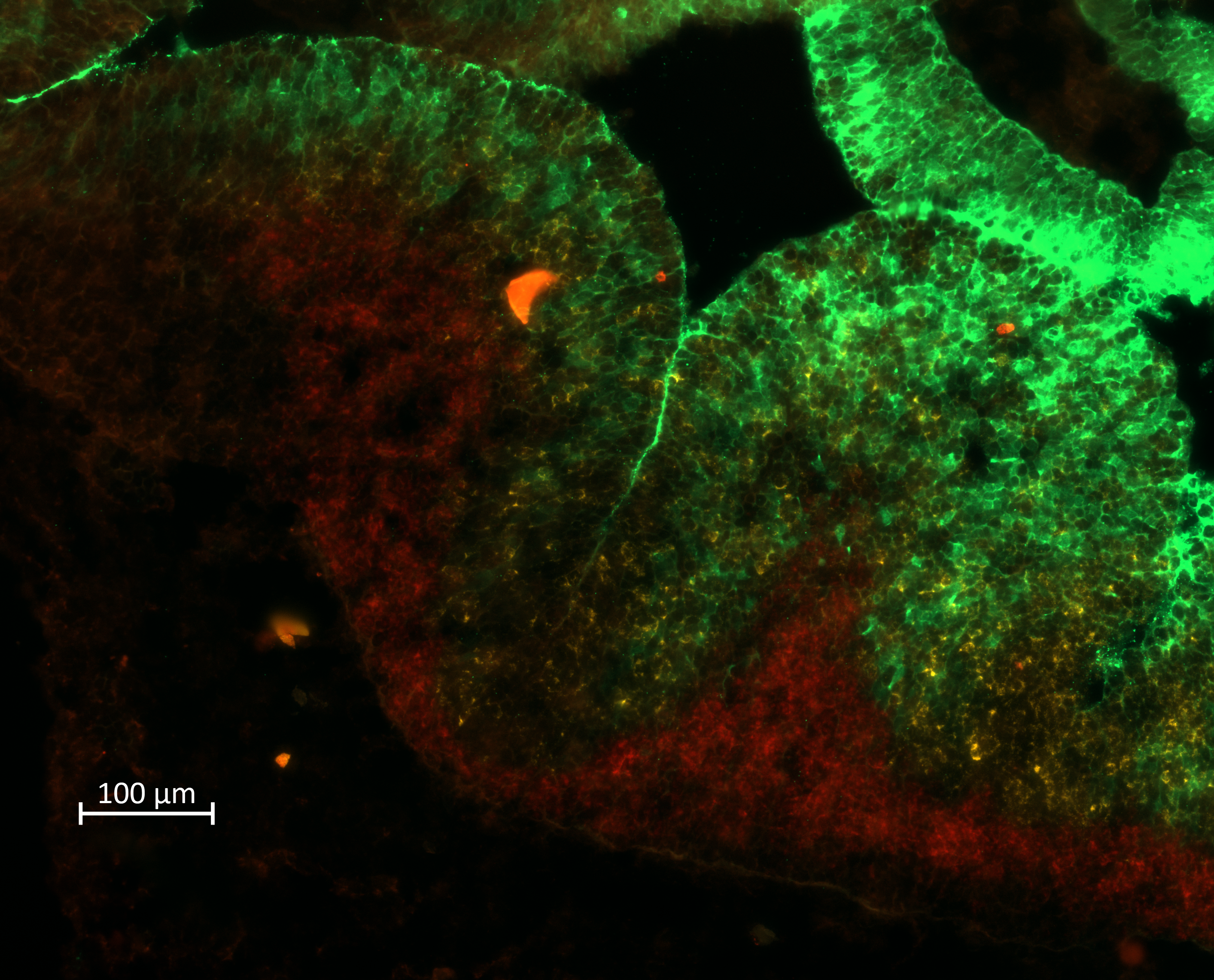

Supplement: Supplementary file 15 — Images for Extended Data Figs. 1, 6 and 7. [file 41593_2025_1999_MOESM15_ESM.zip › image_source/Image_source_ED_Fig1h_Part2.tif]

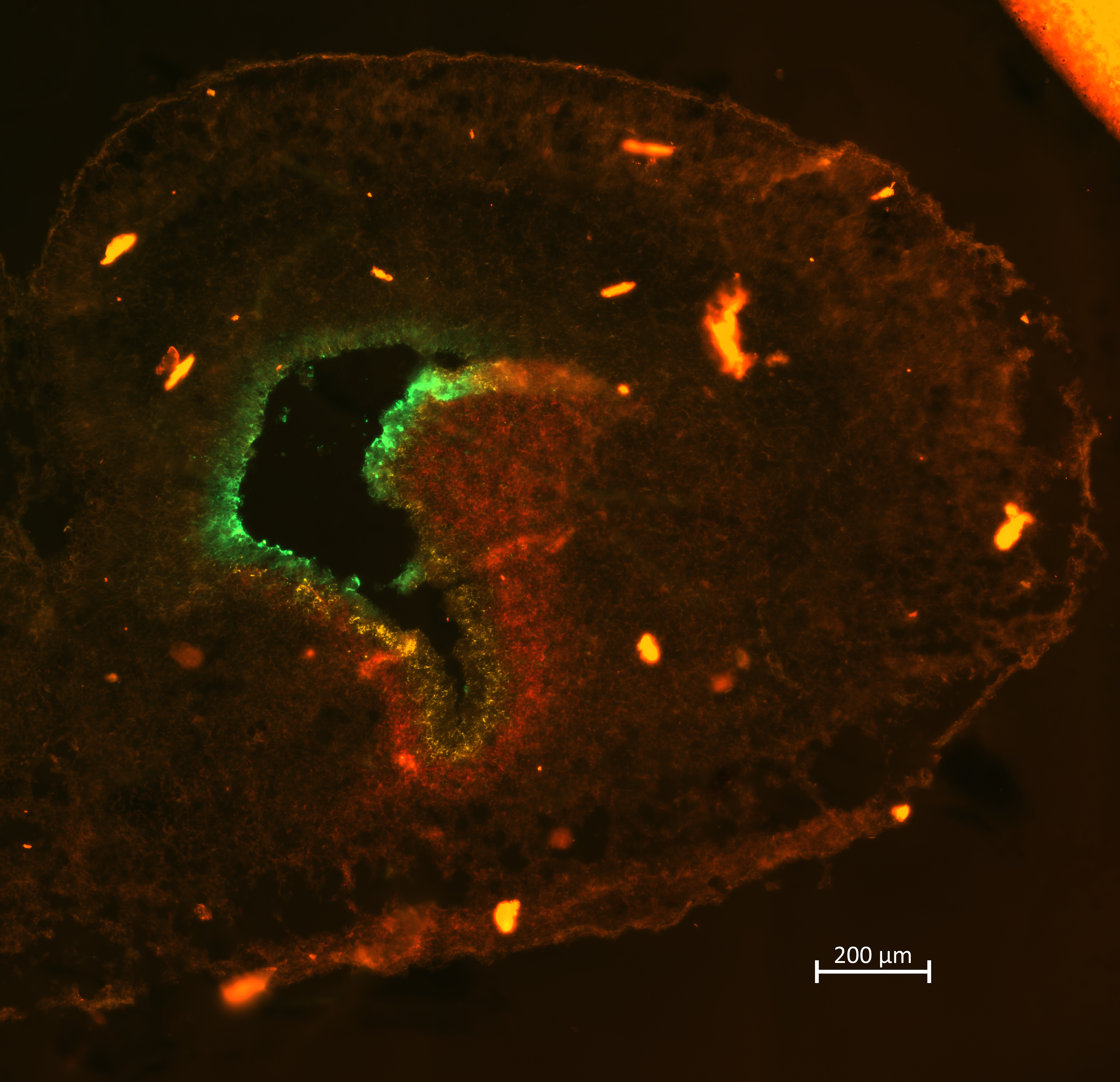

Supplement: Supplementary file 15 — Images for Extended Data Figs. 1, 6 and 7. [file 41593_2025_1999_MOESM15_ESM.zip › image_source/Image_source_ED_Fig1h_Part3.tif]

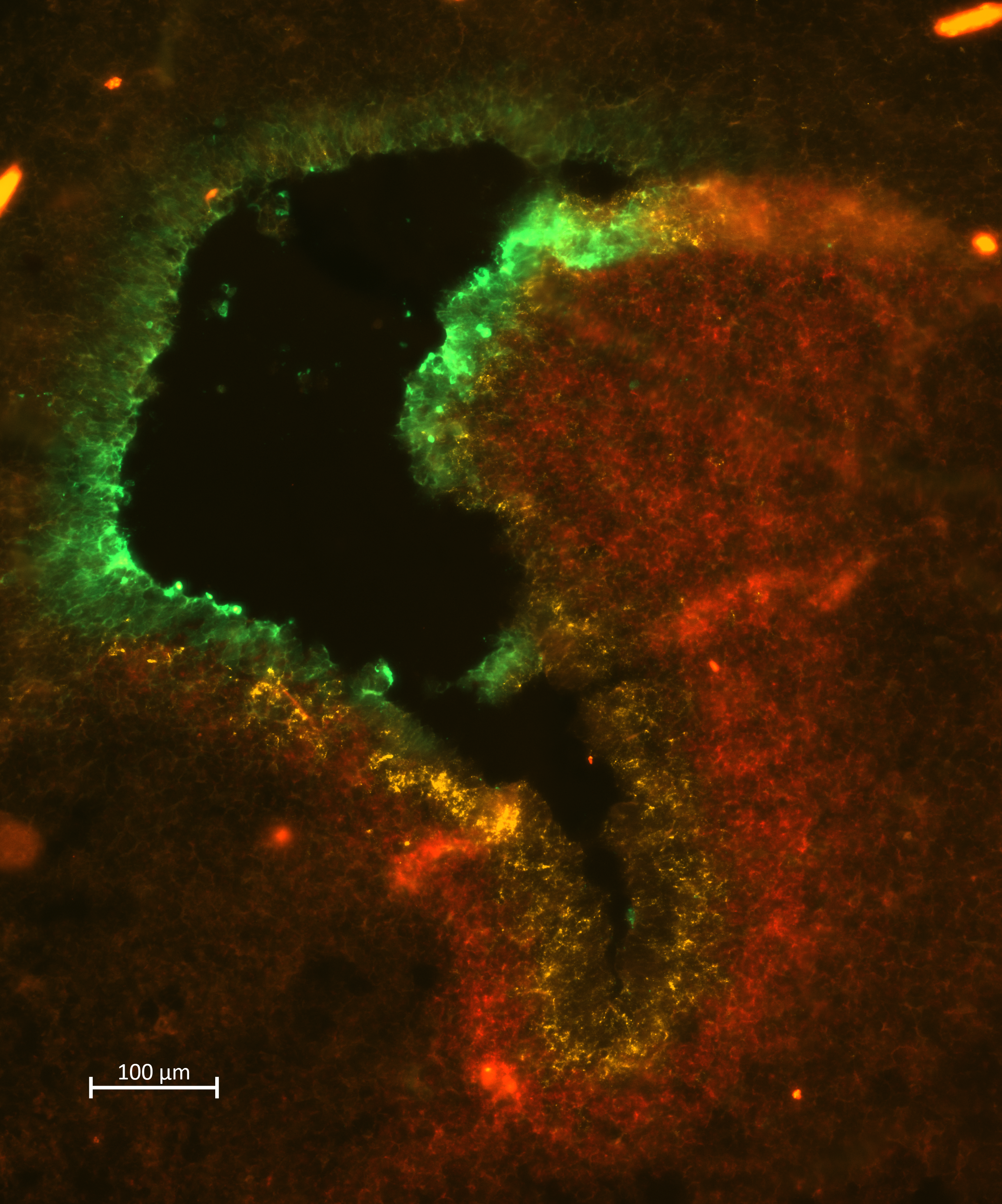

Supplement: Supplementary file 15 — Images for Extended Data Figs. 1, 6 and 7. [file 41593_2025_1999_MOESM15_ESM.zip › image_source/Image_source_ED_Fig1h_Part4.tif]

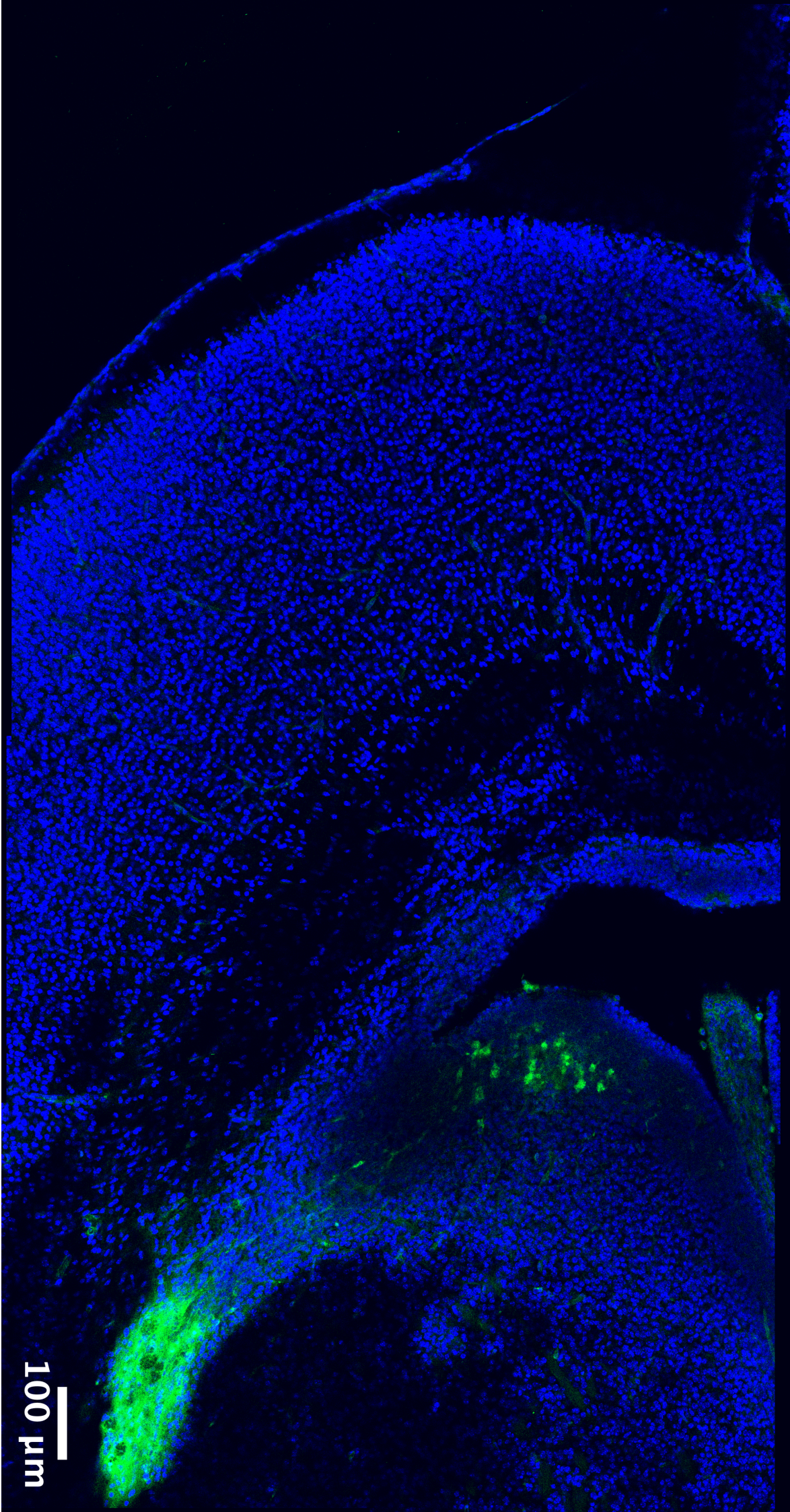

Supplement: Supplementary file 15 — Images for Extended Data Figs. 1, 6 and 7. [file 41593_2025_1999_MOESM15_ESM.zip › image_source/Image_source_ED_Fig6c_E12toE16.pdf]

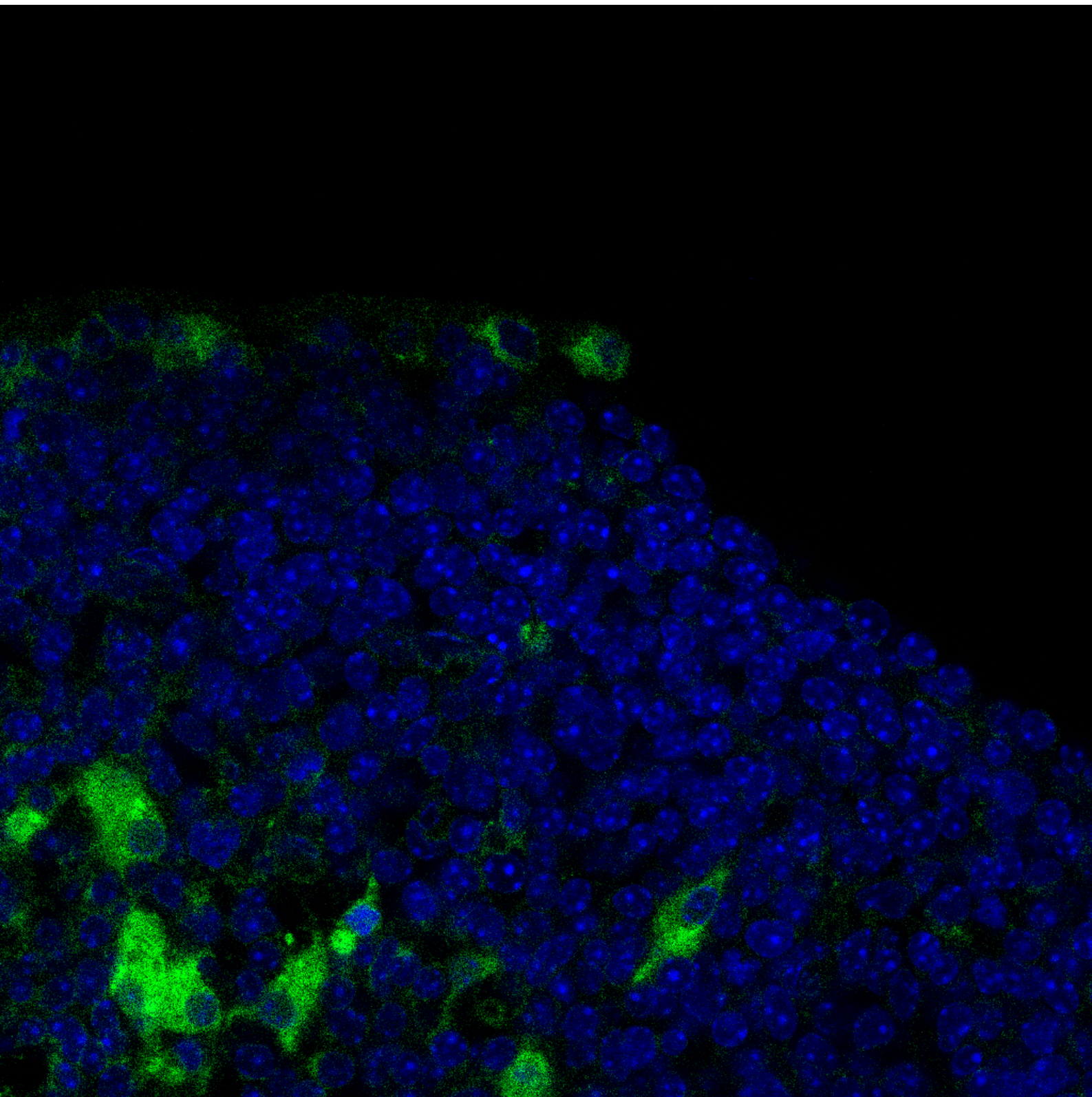

Supplement: Supplementary file 15 — Images for Extended Data Figs. 1, 6 and 7. [file 41593_2025_1999_MOESM15_ESM.zip › image_source/Image_source_ED_Fig6c_E12toE16_63X.pdf]

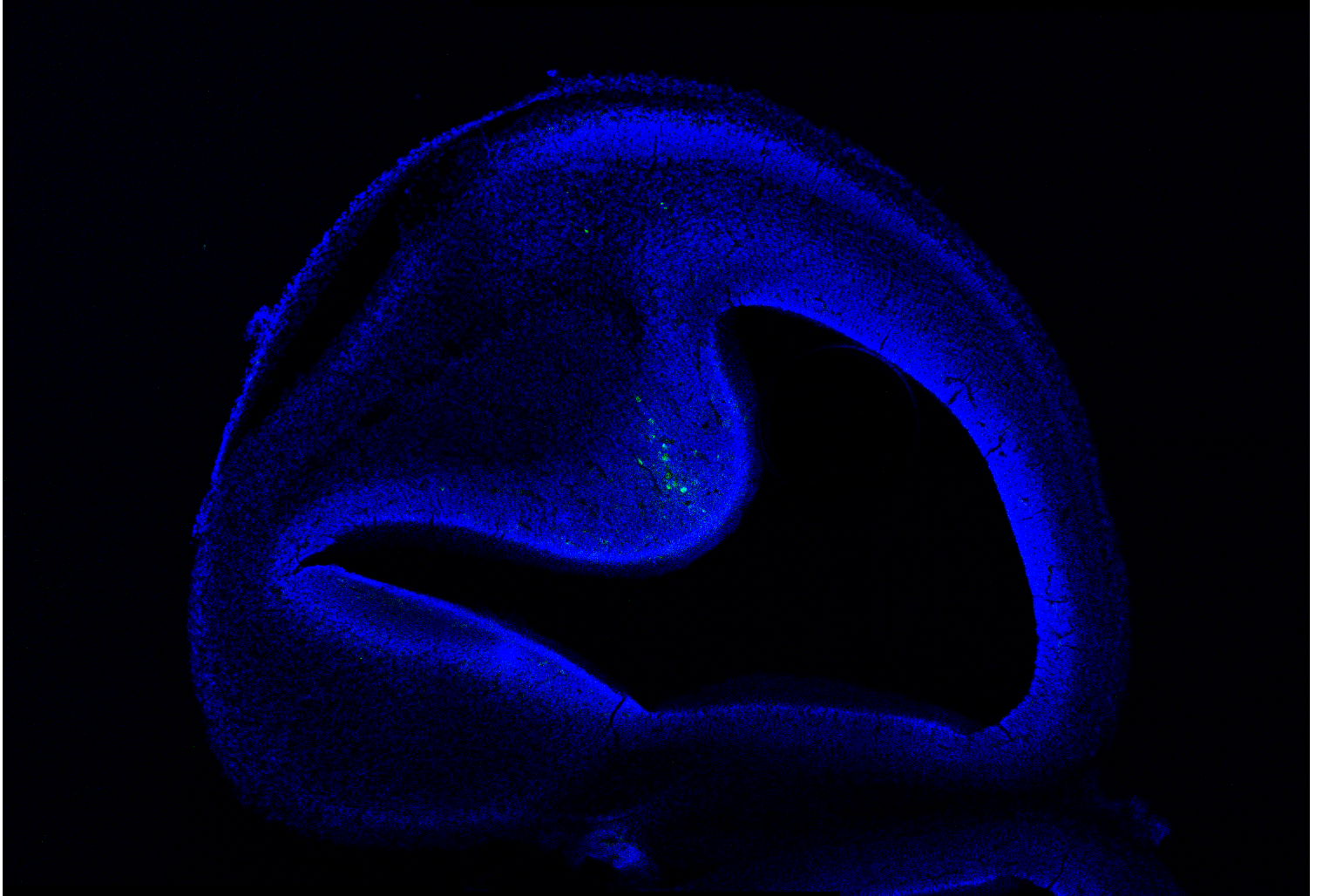

Supplement: Supplementary file 15 — Images for Extended Data Figs. 1, 6 and 7. [file 41593_2025_1999_MOESM15_ESM.zip › image_source/Image_source_ED_Fig6c_E16toE12.pdf]

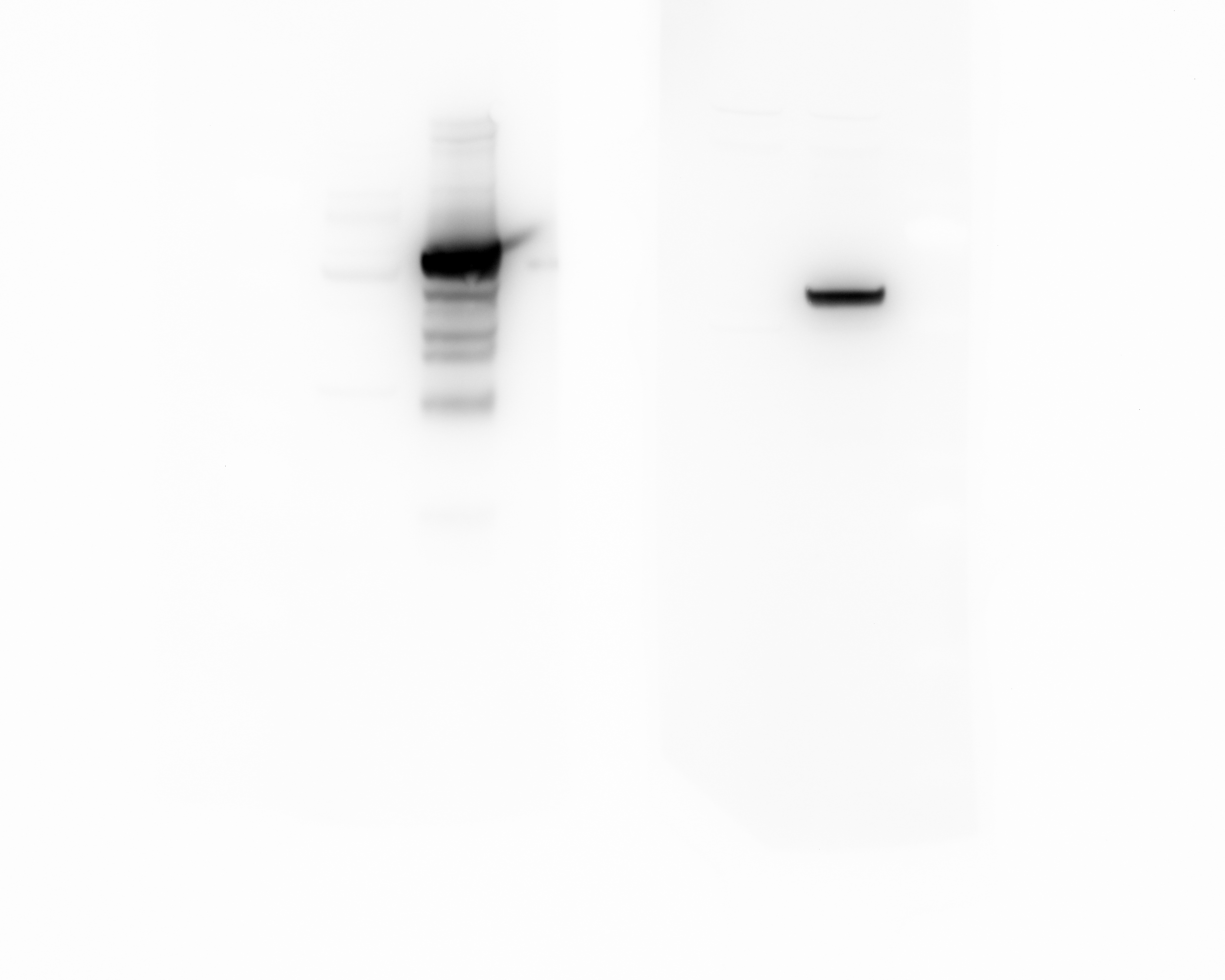

Supplement: Supplementary file 15 — Images for Extended Data Figs. 1, 6 and 7. [file 41593_2025_1999_MOESM15_ESM.zip › image_source/Image_source_ED_Fig7c_Part1.tif]

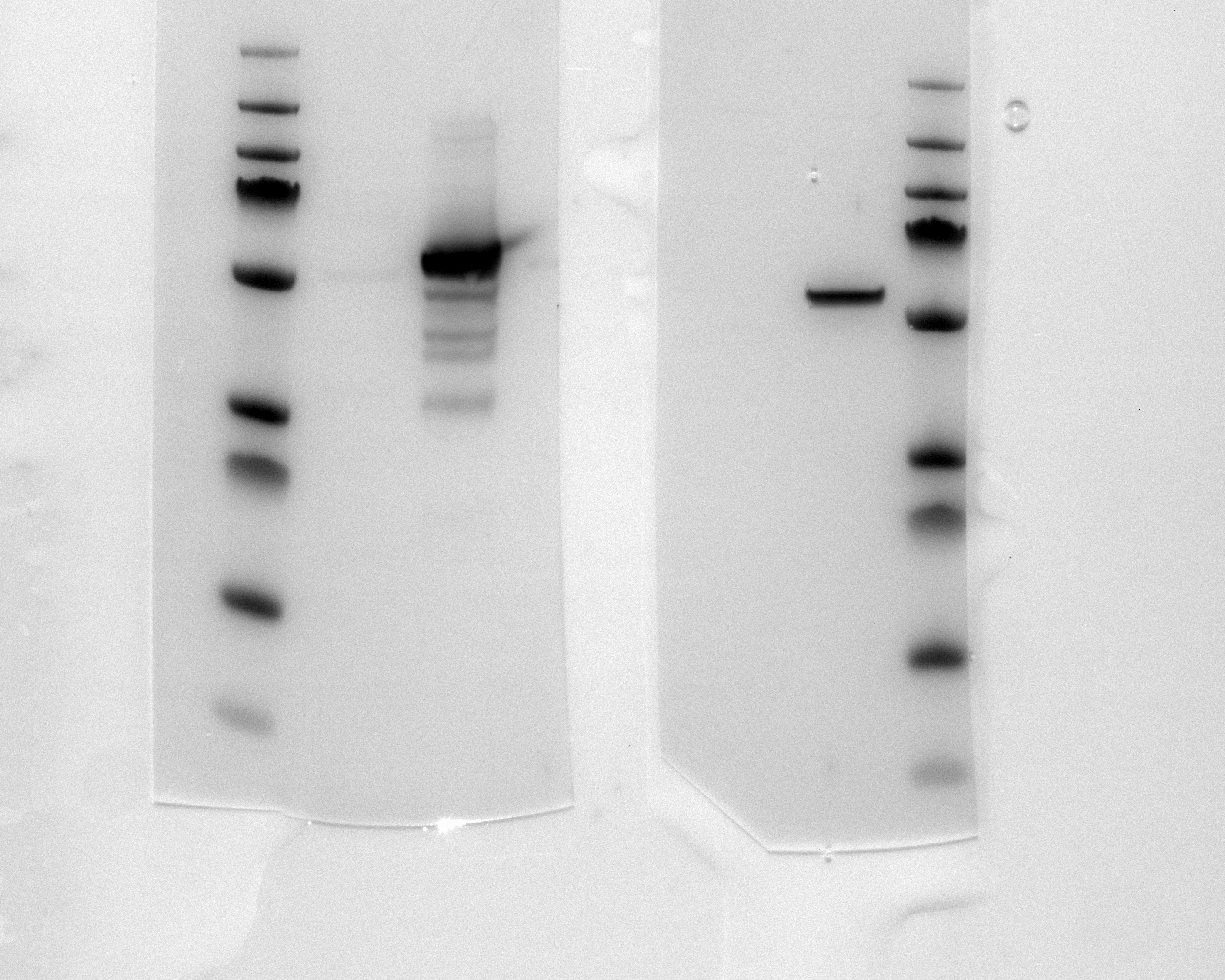

Supplement: Supplementary file 15 — Images for Extended Data Figs. 1, 6 and 7. [file 41593_2025_1999_MOESM15_ESM.zip › image_source/Image_source_ED_Fig7c_Part1_overlay.tif]

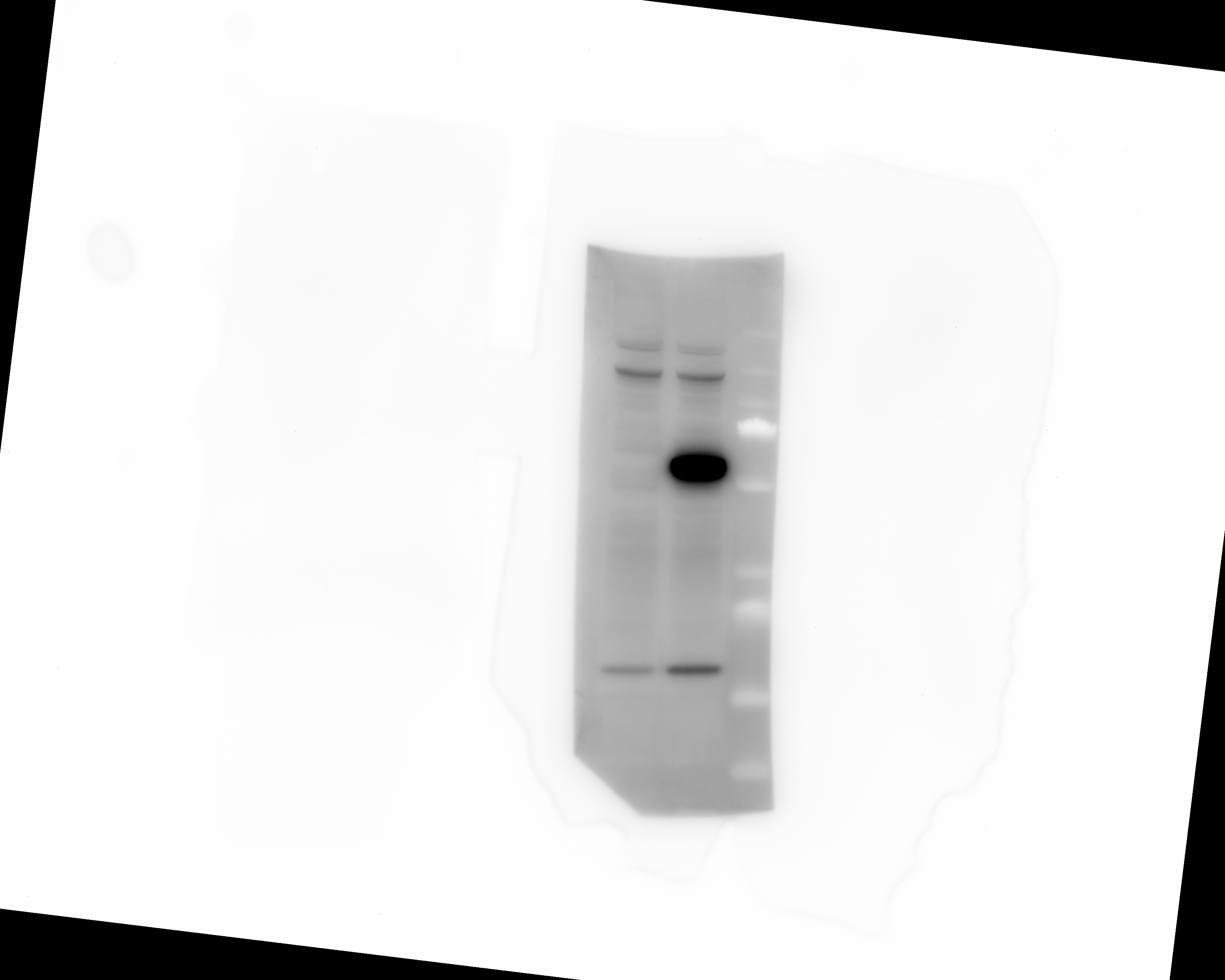

Supplement: Supplementary file 15 — Images for Extended Data Figs. 1, 6 and 7. [file 41593_2025_1999_MOESM15_ESM.zip › image_source/Image_source_ED_Fig7c_Part2.tif]
